# An epitranscriptomic mechanism underlies selective mRNA translation remodelling in melanoma persister cells

**DOI:** 10.1038/s41467-019-13360-6

**Published:** 2019-12-16

**Authors:** Shensi Shen, Sara Faouzi, Amandine Bastide, Sylvain Martineau, Hélène Malka-Mahieu, Yu Fu, Xiaoxiao Sun, Christine Mateus, Emilie Routier, Severine Roy, Laurent Desaubry, Fabrice André, Alexander Eggermont, Alexandre David, Jean-Yves Scoazec, Stéphan Vagner, Caroline Robert

**Affiliations:** 1 INSERM U981, Gustave Roussy Cancer Campus, Villejuif, France; 2 Université Paris-Sud, Université Paris-Saclay, Kremlin-Bicêtre, France; 3 IGF, CNRS, INSERM, University Montpellier, F-34094 Montpellier, France; 4 Institut Curie, PSL Research University, CNRS UMR3348, Orsay, France; 5 Université Paris Sud, Université Paris-Saclay, CNRS UMR3348, Orsay, France; 6 Equipe Labellisée Ligue Nationale Contre le Cancer, Paris, France; 7 Department of Pharmaceutical Chemistry, University of California, San Francisco, California USA; 8 Dermato-Oncology, Gustave Roussy Cancer Campus, Villejuif, France; 9 CNRS-Strasbourg University, UMR7200 Illkirch, France

**Keywords:** Cancer therapeutic resistance, Melanoma

## Abstract

Cancer persister cells tolerate anticancer drugs and serve as the founders of acquired resistance and cancer relapse. Here we show that a subpopulation of BRAF^V600^ mutant melanoma cells that tolerates exposure to BRAF and MEK inhibitors undergoes a reversible remodelling of mRNA translation that evolves in parallel with drug sensitivity. Although this process is associated with a global reduction in protein synthesis, a subset of mRNAs undergoes an increased efficiency in translation. Inhibiting the eIF4A RNA helicase, a component of the eIF4F translation initiation complex, abrogates this selectively increased translation and is lethal to persister cells. Translation remodelling in persister cells coincides with an increased N6-methyladenosine modification in the 5′-untranslated region of some highly translated mRNAs. Combination of eIF4A inhibitor with BRAF and MEK inhibitors effectively inhibits the emergence of persister cells and may represent a new therapeutic strategy to prevent acquired drug resistance.

## Abstract (web summary)

Melanoma persister cells are tolerant to anti-BRAF and anti-MEK inhibition and can trigger cancer relapse. Here the authors show that a subset of N6-methyladenosine modified mRNAs is translationally activated in persister cells. This preferential translation can be abrogated via eIF4A inhibition.

## Introduction

In BRAF^V600E^ mutant melanoma, clinical response to targeted therapy combining BRAF^V600E^ inhibitor (BRAFi) and MEK inhibitor (MEKi) is frequent, with response rates up to 70%. However, the response has limited duration, with half of the patients re-progressing after around 1 year due to drug resistance^1–4^. Multiple mechanisms of resistance, from modification of the drug target to the engagement of a variety of alternative pathways^5^, have been described and are usually explained by the presence of pre-existing rare resistant clones in the tumour cell population or by Darwinian clonal evolution of tumour cells upon drug treatment. In the latter case, tumour cells stochastically acquire drug resistance through genetic mutations under therapeutic selective pressures^6^. Recent studies have shown that a small fraction of cancer cells that survive initial treatment (e.g., anti-epidermal growth factor receptor (EGFR) erlotinib in non-small cell lung cancer) eventually regain their sensitivity to the same drug after a “drug holiday”^7,8^. This reversible drug-tolerant state putatively allows those “persister” cells to survive the initial onslaught of drug before evolving under selective pressure until resistance-conferring permanent genetic mutations emerge. Therefore, eradicating such drug-tolerant cells could potentially stem the torrent of genetic variants that mediate stable resistance to anticancer therapies, turning the persister cell population into an extremely attractive therapeutic target. We previously found that eIF4F-dependent regulation of messenger RNA (mRNA) translation is associated with genetically acquired resistance to BRAFi/MEKi^9^. Although mRNA translation is a fundamental gene expression process^10^, its precise regulatory role in cancer persister cells has not yet been defined.

In this study, we show that BRAF^V600E^ melanoma cells undergoes a reversible mRNA translational reprogramming, by upregulating a subset of mRNAs that encodes epigenetic regulators and mammalian target of rapamycin (mTOR) pathway-related proteins. The upregulation of this subset of mRNAs require the RNA helicase eIF4A, whose inhibition selectively kills the melanoma persister cells. These mRNAs specifically harbour N6-methyladenosine (m^6^A) modification in their 5′-untranslated regions (5′-UTRs) and accordingly knockdown of the m^6^A methylase complex proteins, including METTL3 or WTAP, can abrogate their association with polysomes. In addition, inhibition of eIF4A decreases the association of m^6^A-modified mRNAs with polysomes and prevents the emergence of BRAFi/MEKi-resistant clones.

## Results

### Translation activity is altered in melanoma persister cells

**Fig. 1:**
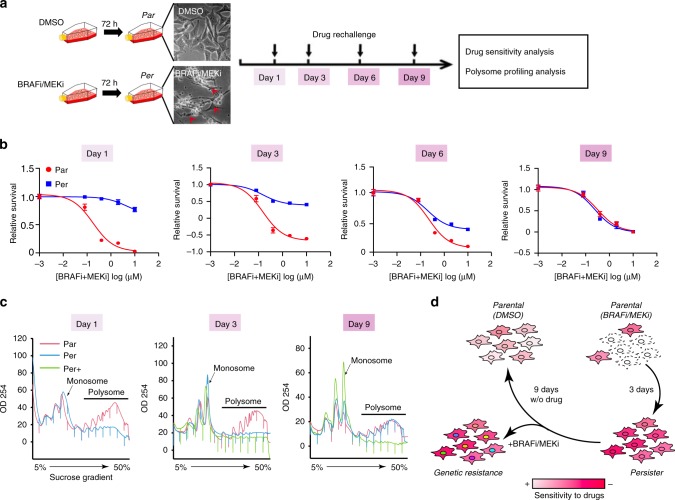
Melanoma persister cells have altered translation activity. **a** Schematic of melanoma persister cell generation and subsequent analyses. Par: parental cells treated with dimethylsulfoxide (DMSO), Per: persister cells marked as red arrows. **b** Reversible drug tolerance of persister cells upon BRAFi/MEKi removal. Persister and parental cells were re-challenged with BRAFi/MEKi treatment on the indicated days and drug sensitivity was analysed by WST-1-based cell viability assay. **c** Polysome profiles of persister cells at various time points, assessed by 5–50% sucrose-gradient ultracentrifugation. Per+ represents persister cells upon continuous BRAFi/MEKi exposure. **d** Schematic view shows that persister cells re-acquire similar drug sensitivity as parental cells upon BRAFi/MEKi removal. Upon continuous drug exposure, persister cells can serve as a reservoir for the development of genetic acquired resistant cells. The raw data of **b** are available in Source Data.

To study the underlying mechanisms of this phenomenon, we first analysed survival of BRAF^V600E^ mutant A375 melanoma cells in the presence of lethal concentrations of BRAFi (PLX4032) and MEKi (cobimetinib). Tested cell populations (99.7%) contained surviving persister cells (Supplementary Fig. 1a). This is similar to those described in the context of lung cancer cells treated with EGFR inhibitors^11^. One hundred per cent of tested single cell-derived sub-clones (*n* = 5) contained surviving persister cells (Supplementary Fig. 1a and Methods), excluding pre-existing genetic heterogeneity. These results indicate that the persistent state represents a major survival mechanism against BRAFi/MEKi treatment in melanoma. Indeed, compared with parental A375 cells (Par) that had never been exposed to the treatment, persister cells (Per) showed strong tolerance to a second challenge of BRAFi/MEKi treatment applied 1 day after treatment withdrawal as shown by viability (Fig. 1a, b) and caspase-3/7 activity assays (Supplementary Fig. 1b). This tolerance was not associated with mitogen-activated protein kinase (MAPK) pathway re-activation as revealed by the analysis of ERK1/2 phosphorylation (Supplementary Fig. 1c). Withdrawal of BRAFi/MEKi resulted in a progressive re-acquisition of drug sensitivity similar to that of parental cells over a period of 9-day drug-free culture (Fig. 1b). This reversibility of drug tolerance was also observed in two other BRAF^V600E^ mutant cell lines WM983B and Malme-3M (Supplementary Fig. 1d). Together, these results indicate an adaptive, non-genetic mechanism underlying the establishment of persister cells.

To explore whether melanoma persister cells ultimately give rise to diverse genetic resistant clones, we cultured single cell-derived persister cell populations in the presence of escalating concentrations of BRAFi/MEKi for ~3 months (Supplementary Fig. 2a, b). Double-resistant (DR) clones (A375DR) were then confirmed for their stable, irreversible resistance to BRAFi/MEKi after ~10 passages in a drug-free medium by dose-dependent sensitivity assays (Supplementary Fig. 2c) and clonogenic assays (Supplementary Fig. 2d). Persister-derived DR clones showed BRAF alternative splicing alterations and BRAF-MAPK pathway re-activation for three of them (DR5, DR6 and DR7; Supplementary Fig. 3a, b). Whole-exome sequencing (WES) revealed a diverse panel of genetic events including some known to be associated with melanoma resistance (Supplementary Fig. 3c, d). This confirms that the persister cells are capable of developing distinct genetic resistant cells. Thus, understanding the potential non-genetic regulatory mechanisms in persister cells may identify therapeutically exploitable vulnerabilities of these cells and prevent the development of genetically acquired resistance.

We explored the role of translational control in persister cells by western blotting analysis of puromycin incorporation into nascent proteins as a representation of protein synthesis. There was a global reduction of protein neo-synthesis in persister cells compared with parental cells (Supplementary Fig. 4a). To investigate whether the translational changes were associated with the persistent state, we studied the polysome profiles of parental and persister cells cultured in the presence (Per+) (i.e., continued presence of persister cells in the population) or absence (Per) (i.e., loss of persister cells from the population) of BRAFi/MEKi. On day 1, persister cell lysates, separated by ultracentrifugation on a sucrose gradient, had a lower content of polysome-bound mRNAs than that of the parental cells (Fig. 1c), consistent with their lower protein synthesis. Persister cells regained the same translation activity as parental cells after 9 days of culture in the absence of BRAFi/MEKi (Fig. 1c). However, in the continued presence of BRAFi/MEKi, the translation activity of persister cells (Per+) remained at the same reduced level (Fig. 1c). Together, these results suggest that melanoma persister cells undergo a reversible reduction in mRNA translation activity coinciding with their reversible tolerance to BRAFi/MEKi upon withdrawal of BRAFi/MEKi, whereas they can further evolve towards genetic resistance in the continued presence of BRAFi/MEKi treatment (Fig. 1d).

### A subset of actively translated mRNAs drives persistence

**Fig. 2:**
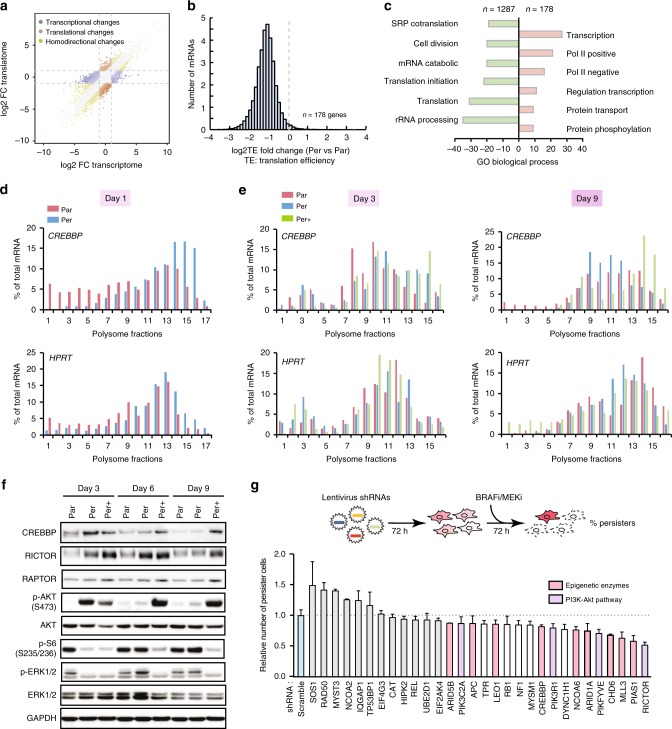
Dynamic translation remodelling drives melanoma persistence. **a** Transcriptional and translational analysis of genome-wide polysome profile between persister and parental cells. Colour-coded are genes with the adjusted *P*-value < 0.05 with fold change >2 (DESeq2); *n* = 3 per group for both polysome profiling and exon-array. One hundred and seventy-eight transcripts showed upregulation at the translational level. **b** Differential translational efficiency (TE = Expression_polysome mRNA_/Expression_total mRNA_) of persister vs. parental cells. **c** David gene ontology (GO) analysis of mRNAs regulated at the translational level (red: upregulation; green: downregulation) in persister vs. parental cells. **d**, **e** RT-qPCR quantification of *CREBBP* mRNA (top panel) or *HPRT* mRNA (bottom panel) in fractions (horizontal axes) obtained by sucrose-gradient ultracentrifugation of lysates from persister versus parental cells from day 1 (**d**), day 3 and 9 (**e**) following BRAFi/MEKi withdrawal. Par: parental cell; Per: persister cell cultured in drug-free medium; Per+: persister cell cultured in BRAFi/MEKi containing medium. **f** Protein level and related pathway activity analysis by western blotting at various time points. S: serine. **g** Lentivirus-based shRNA screening for persister cell survival. A375 cells were transduced with pLKO.1 lentivirus shRNAs for 3 days and then were treated with lethal concentrations of BRAFi/MEKi (both at 1 μM) for 3 days. Percentage of survival persister cells was evaluated by WST-1-based cell viability assay, data were normalized to the percentage of persister cells from scramble shRNA-transduced cells. The raw data of **d**, **e**, **g** and **f** are available in Source Data.

To further investigate the role of mRNA translation in persister cells, we performed genome-wide polysome profiling analyses to identify translationally regulated mRNAs (Supplementary Fig. 4b). By conducting matched exon-array-based transcriptome and translatome analyses, we classified mRNAs in three groups (Supplementary Data 1 and 2): group 1 with at least a twofold change in the level of cytoplasmic mRNAs, but less than twofold change in the level of polysomal mRNAs (Fig. 2a, blue points); group 2 with at least a twofold change in the level of polysomal mRNAs but less than twofold change in the level of cytoplasmic mRNAs (Fig. 2a, orange points); group 3 with at least a twofold change, either increase or decrease, in the level of both cytoplasmic and polysomal mRNAs (Fig. 2a, green points). We focused our study on the mRNAs from groups 2 and 3, for which the mRNAs levels are significantly different in polysomal fractions vs. cytoplasmic fractions, reflecting translational regulation (Fig. 2a and Supplementary Fig. 4c-e). Gene Ontology analysis showed that the translationally downregulated mRNAs (*n* = 1287) are predominantly involved in cell division and mRNA translation, which corresponds to a quiescent cell state that was already observed in lung cancer persister cells^11,12^. In contrast, 178 mRNAs were more efficiently translated in persister cells, in spite of the overall reduction of translation efficiency (TE) compared with that of parental cells (Fig. 2a, b). These 178 mRNAs encode proteins associated with transcription regulation and intracellular signalling (Fig. 2c and Supplementary Fig. 5a). STRING network analysis showed that these 178 mRNAs are enriched in an intensively wired network comprising histone posttranscriptional modifiers^13–15^ (e.g., CREBBP, MLL3 and NCOA6), readers^16,17^ (e.g., TP53BP1, BPTF and DHX36), chromatin remodellers^18,19^ (e.g., CHD6, ARID5B and ARID1A) and stress-responsive kinases^20–22^ (e.g., HIPK1, EIF2AK4 and RICTOR) (Supplementary Fig. 5b). We validated the polysome profiling results by performing quantitative reverse transcriptase PCR (RT-qPCR) on a subset of these mRNAs and confirmed that they were enriched in the heavy polysome (actively translating) fractions (Fig. 2d and Supplementary Fig. 6).

Low translation activity was previously shown to maintain tumour stem cell-related quiescent state, but certain mRNAs maintained their TE to support cell survival in response to cytotoxic stress in a *K5-Sos*/*Nsun2*^−/−^ model^23^. We hypothesized that mRNAs deviating from global translational reduction may also play a role in persister cell survival. We thus explored whether the dynamics of this subset of translationally upregulated mRNAs in persister cells correlated with the persistent state upon BRAFi/MEKi treatment. We performed RT-qPCR on the mRNAs extracted from sucrose gradient fractions of persister cells in the presence (Per+) or absence (Per) of drugs. After 9 days of culture without BRAFi/MEKi, these translationally upregulated mRNAs regained their baseline TE, similar to that observed in the parental cells (Fig. 2e and Supplementary Figs. 6 and 7a). In accordance, protein levels of the translationally upregulated mRNAs, such as CREBBP and RICTOR, also returned back to their basal levels, consistent with the reversible phosphorylation of AKT seen upon BRAFi/MEKi withdrawal (Fig. 2f). This was also coupled with the re-phosphorylation of ERK1/2 and S6 ribosomal protein that was completely inhibited in persister cells (Fig. 2f), reflecting a restoration of BRAF-MAPK pathway activity. However, these mRNAs remained more efficiently translated in the continuous presence of BRAFi/MEKi (Fig. 2e and Supplementary Fig. 7a). Notably, phosphorylation of AKT was also sustained during the 9-day period in the presence of BRAFi/MEKi (Fig. 2f).

To determine whether the translationally upregulated mRNAs were involved in persistence, we transduced A375 melanoma cells with lentivirus expressing short hairpin RNAs (shRNAs) targeting the 30 top-ranked mRNAs based on their degree of network connectivity in the STRING network analysis (Supplementary Fig. 5b). Cells transduced with each shRNA were exposed to BRAFi/MEKi for 72 h and the percentages of residual persister cells were measured by the WST-1 cell viability assay, and compared with cells harbouring shRNAs without treatment (Fig. 2g and Supplementary Fig. 7b), the percentage of residual persister cells in each experiment was subsequently normalized to that of cells transduced with a scrambled shRNA. This analysis revealed that knockdown of 18 genes (unpaired non-parametric Kolmogorov–Smirnov *t*-test, *p* < 0.01) further reduced persister cell survival significantly beyond the control. Of these, (1) nine genes encode epigenetic-related enzymes, such as histone acetyltransferase CREBBP and methyltransferase MLL3 that are involved in enhancer-related transcription activation^13,15^, or nucleosome remodellers, such as CHD6, ARID5B and ARID1A; and (2) four genes are involved in the PI3K-mTORC2 pathway, including RICTOR, PIKFYVE and PIK3R1 (Fig. 2g). We further explored the role of the PI3K-mTORC2 pathway in persister cell survival. We observed increased RICTOR expression (Supplementary Fig. 7c) and elevated levels of phosphorylated mTOR at serine 2481 and AKT at serine 473 in persister cells (Supplementary Fig. 7d). Serine 2481 is a marker of mTORC2 activation, which phosphorylates AKT^24^. Phosphorylation of mTOR at serine 2448 was also increased in persister cells, but to a lesser extent compared with parental cells (Supplementary Fig. 7d). This increase in persister cells may result from AKT activation, as this site is PI3K/AKT-dependent^25,26^. Accordingly, PP242 (a mTORC1 and mTORC2 ATPase inhibitor) abrogated both phosphor-AKT (S473) and phosphor-mTOR (S2481), whereas rapamycin^27^ (an allosteric inhibitor of mTORC1) inhibited the phosphorylation of mTOR Ser 2448 only in persister cell (Supplementary Fig. 7d). All together, these findings support our model that melanoma persister cells undergo drug-dependent reversible mRNA translation remodelling, and that a subset of translationally upregulated mRNAs coding for proteins involved in multiple regulatory pathways is associated with persister cell survival.

### EIF4A inhibition selectively kills melanoma persister cells

**Fig. 3:**
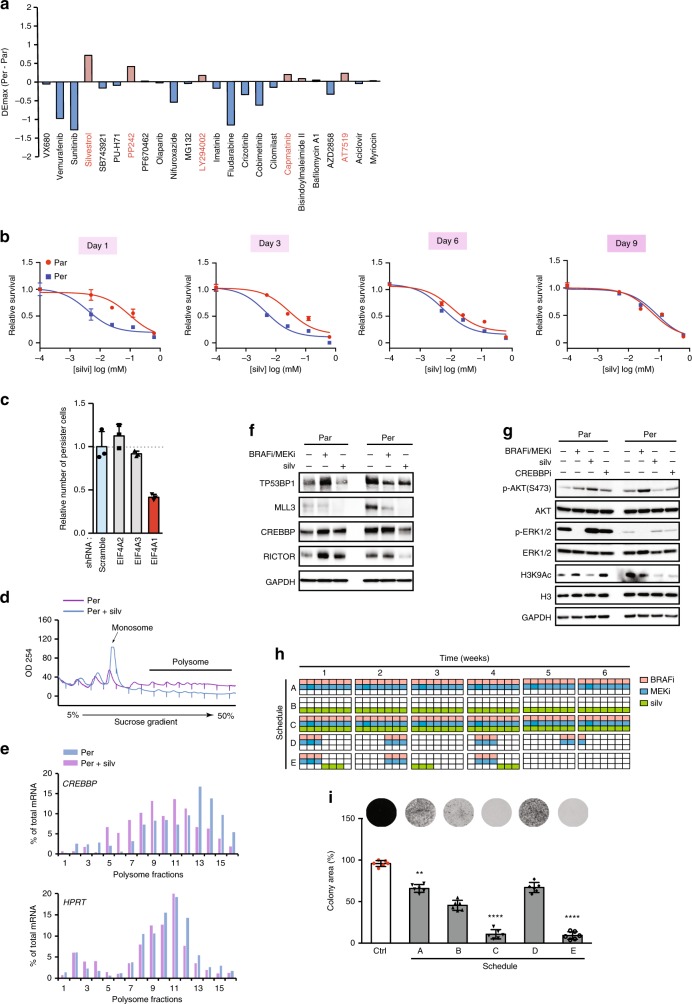
Targeting translation remodelling by eIF4Ai eradicates persister cells. **a** Drug sensitivity of persister and parental cells to a small panel of inhibitors. The cells were treated for 48 h and cell viability was assayed using WST-1. Δ*E*_max_: differential maximum effect between persister and parental cells. **b** Reversible sensitivity of persister cells to silvestrol (silv). Persister and parental cells were re-challenged by silvestrol treatment on indicated days; the drug sensitivity was analysed by WST-1-based cell viability assay. **c** Lentivirus-based shRNA knockdown of eIF4A1, eIF4A2 and eIF4A3 for 3 days and then cells were treated with lethal concentrations of BRAFi/MEKi (both at 1 μM) for 3 days. Percentage of survival persister cells was evaluated by WST-1-based cell viability assay. Data were normalized to the percentrage of persister cells from scramble shRNA-transduced cells. **d**, **e** RT-qPCR quantification of *CREBBP* mRNA or *HPRT* mRNA in fractions obtained by sucrose-gradient ultracentrifugation of lysates from persister cells in the presence or absence of silvestrol (silv). Polysome profiles (**d**) and RT-qPCR histogram (**e**) were displayed. **f** Western blotting analysis of the effect of silvestrol (silv) on candidate mRNAs that were regulated at the translational level in persister vs. parental cells. Cells were treated with 30 nM silvestrol (silv) or 1 μM BRAFi/MEKi for 8 h. **g** Western blotting analysis of the effect of silvestrol (silv) on the activity of the mTORC2-AKT pathway and histone modifications in persister versus parental cells. Cells were treated with 30 nM silvestrol (silv) or 1 μM BRAFi/MEKi for 8 h. **h**, **i** Combination of silvestrol (silv) and BRAFi/MEKi abrogates persister cell-derived colony formation. A schematic representation of the drug combination treatment schedules (**h**) and their effect on the clonogenic assay of persister cells are presented (**i**) (*n* = 6, *p*-value < 0.0001, unpaired *t*-test). The raw data of **b**, **c**, **e**, **f**, **g** and **i** are available in Source Data.

In an attempt to target persister cells as a therapeutic approach, we screened a panel of small-molecule compounds that target different kinases or proteins known to be involved in cancer resistance as well as various inhibitors of the pathways that were found to be upregulated at the translational level. These data showed that silvestrol, an inhibitor of the eIF4A RNA helicase component of the eIF4F translation initiation complex, was the most selectively lethal compound towards melanoma persister cells (Fig. 3a, Supplementary Fig. 8a and Supplementary Data 3). A similar selective sensitivity to silvestrol was also observed in PC9 non-small cell lung cancer persister cells (Supplementary Fig. 8b), another well-characterized persister cell model. We further evaluated the sensitivity of melanoma persister cell to three other translation initiation inhibitors, including 4E1RCat, a specific inhibitor that disrupts the eIF4E–eIF4G interaction in the eIF4F complex; hippuristanol, a compound that prevents eIF4A from binding to mRNA; and pateamine A, an inhibitor that leads to depletion of eIF4A from the eIF4F complex. All of them showed stronger toxicities on the persister cells than on the parental cells (Supplementary Fig. 8c). The sensitivity of persister cells to silvestrol was reversible upon BRAFi/MEKi withdrawal (Fig. 3b), underscoring its close correlation with the persistent state. Consistently, targeting translation remodelling directly through eIF4A inhibition was notably much more effective as compared with the effects obtained when targeting translationally upregulated pathways, such as CREBBP (CREBBPi)^28^, H3K27m3 demethylase (KDM6i)^29^ or mTORC2 inhibitor (PP242) (Supplementary Fig. 8d).

Three eIF4A proteins have been characterized in vertebrates, including eIF4A1, eIF4A2 and eIF4A3. eIF4A1 is an ATP-dependent DEAD-box RNA helicase that assists in unwinding secondary structures within the 5′-UTR of mRNAs to allow ribosome scanning^30^. Although eIF4A1 and eIF4A2 show ~90% sequence identity, they are not functionally redundant in vivo. For instance, eIF4A1 knockdown, which is known to induce an increase in eIF4A2, leads to a decrease of [^35^S] methionine incorporation and global mRNA distribution in polysome profiles^31^, showing that the upregulation of eIF4A2 does not compensate for the reduction in mRNA TE upon eIF4A1 depletion. In addition, eIF4A1 and eIF4A2 have distinct binding partners, eIF4A1 predominantly binds to eIF4G^32^ while eIF4A2 preferentially binds to cNOT7 (a member of the CCR4-NOT complex)^33^. eIF4A3 is functionally distinct from eIF4A1 and eIF4A2, despite sharing 60% homology^34^. eIF4A3 is likely not involved in translation control, as it principally resides in the nucleus where it forms a key component of the exon junction complex and plays a major role in the nonsense-mediated mRNA decay^35^. We found that knockdown of eIF4A1, but not eIF4A2 or eIF4A3, effectively inhibited the emergence of persister cells in the presence of BRAFi/MEKi (Fig. 3c). In addition, we observed reduced levels of eIF4A1 and ribosomal proteins in persister cells (Supplementary Fig. 8e). Of note, this situation is similar to that seen in a genetic blood disorder, where a limited ribosomal pool selectively disrupts translation of specific transcripts and affects lineage commitment^36^. It is possible that persister cells are more sensitive to the inhibition of eIF4A1 because the limited levels of translation machinery components are re-allocated to the selective translation of mRNAs that are critical for persister cell survival.

We next explored the effect of eIF4Ai on the translationally upregulated mRNAs in melanoma persister cells by performing polysome profile-based RT-qPCR assays with or without silvestrol (Fig. 3d, e). We observed a shift of these mRNAs from heavy polysomes to monosomes in the presence of silvestrol in persister cells (Fig. 3e and Supplementary Fig. 8f). In accordance, silvestrol efficiently decreased the protein levels of the mRNAs that were upregulated in persister cells (Fig. 3f). We tested the effect of silvestrol on the activation of the two processes translationally upregulated in persister cells, i.e., mTORC2 pathway and epigenetic regulation. Although silvestrol showed no effect on MAPK pathway activity, it specifically decreased phosphor-AKT activation in persister cells (Fig. 3g), which is consistent with the decreased expression of RICTOR observed upon eIF4A inhibition^37^ (Supplementary Fig. 7e). Silvestrol also decreased H3K9Ac levels in persister cells in a similar manner as what was observed with CREBBPi (Fig. 3g). These results suggest that silvestrol can simultaneously inhibit the multiple regulatory pathways involved in translation remodelling in melanoma persister cells. This could overcome the challenges of eradicating persister cells by using a combination of several agents targeting multiple translationally upregulated downstream pathways.

We hypothesized that eIF4A inhibition in combination with BRAFi/MEKi may meaningfully improve outcomes of current targeted therapy in BRAF^V600^ melanoma, by directly targeting residual persister cells^38^. We thus evaluated the efficacy of silvestrol combined with BRAFi/MEKi in preventing persister cell colony formation, by comparing five treatment strategies (Fig. 3h). As BRAFi-dependent fitness of melanoma cells has been previously modelled, leading to the proposition of an intermittent BRAFi regimen to maximize the duration of patient response^7^, we tested several regimens with BRAFi/MEKi given continuously or intermittently (schedules A and D, respectively). This was compared with silvestrol given as a monotherapy (schedule B) or combined with BRAFi/MEKi continuously or sequentially (schedules C and E, respectively). Schedules A, B and D were found to be much less effective to prevent colony formation than when silvestrol and BRAFi/MEKi were given in combination, either continuously (schedule C) or sequentially (schedule E) (Fig. 3i). Indeed, with these two latter schedules, we observed a strong anti-clonogenic effect, with a complete inhibition of colony formation (Fig. 3i). These results suggested that eIF4A inhibition, in combination with BRAFi/MEKi, either impeded the transition of parental cells towards persister cells or selectively targeted persister cells, and that combining eIF4A inhibition with BRAFi/MEKi could be a promising strategy to overcome the challenge of targeting heterogeneous melanoma cell populations and eventually abrogate the emergence of resistant cells.

### m^6^A is enriched on polysomal mRNAs in persister cells

**Fig. 4:**
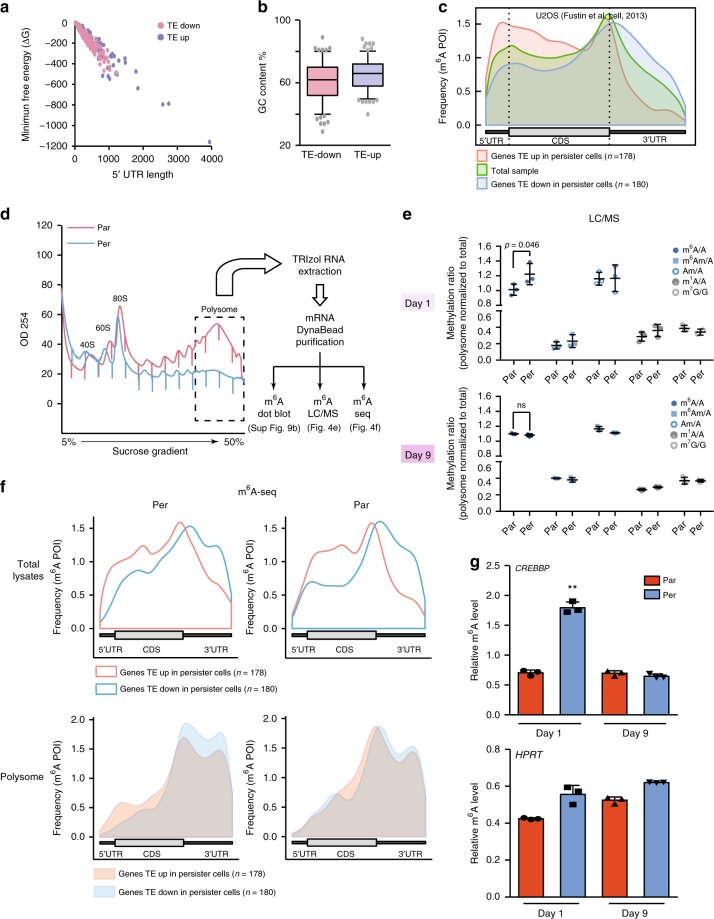
N6-methyladenosine (m^6^A) is enriched in the mRNAs translationally upregulated in persister cells. **a** 5′-UTR minimum free energy and length of the mRNAs translationally upregulated and top 180 downregulated in persister cells. **b** GC content of the mRNAs translationally upregulated and top 180 downregulated in persister cells. TE: translation efficiency. **c** The distribution of m^6^A peaks for mRNA upregulated (red) and downregulated (blue) at the translational level in persister cells. The whole population of mRNAs was plotted in green as a control. **d** Heavy polysome-bound mRNAs were extracted and purified for m^6^A dot plot assay, m^6^A LC/MS-MS assay and m^6^A RNA immunoprecipitation followed by RNA sequencing (m^6^A-seq) in persister vs. parental cells. **e** Liquid chromatography–mass spectrometry (LC/MS-MS) nucleoside modification analysis. Total RNAs were purified by poly(A) enrichment and were subjected to digestion. Triplicate samples were then subjected to LC/MS-MS quantification. Each type of RNA methylation was first normalized to non-methylated nucleotide base (A or G) and polysome RNA methylation was then normalized to total RNA methylation (input). m^6^A: 6-methyladenosine; m^6^A_m_: N^6^,2’-*O*-dimethyladenosine; A_m_: 2’-*O*-methyladenosine; m^1^A: 1-methyladenosine; m^7^G: 7-methylguanosine; A: adenosine; G: guanosine (*n* = 3, unpaired *t*-test, ns: nonsignificant). **f** Metagene profiles of enrichment of m^6^A modifications across mRNAs corresponding to translationally upregulated transcripts and the top 180 translationally downregulated transcripts in parental and persister cells. Top panel, metagene profiles of m^6^A modifications of total lysate from parental or persister cells; bottom panel: metagene profiles of m^6^A modifications of heavy polysome fractions from parental or persister cells. CDS, coding sequence. **g** RT-qPCR quantification of m^6^A enrichment in polysome-bound *CREBBP* mRNA and *HPRT* mRNA at indicated time points (*n* = 3, **p-value < 0.01, unpaired *t*-test). The raw data of **e** and **g** are available in Source Data.

We next explored potential mRNA features that might be involved in the selective translation observed in persister cells. As the 5′-UTR is crucial for mediating translational control of eukaryotic mRNAs in terms of the length and secondary structures^39^, we first interrogated the 5′-UTR features of the 178 mRNAs identified in our polysome profiling analysis (Fig. 2a) in comparison with those from the top 180 translationally downregulated transcripts. We did not observe any significant differences in terms of the length or the minimum free energy of the corresponding 5′-UTRs (Fig. 4a). In addition, the GC content in the 5′-UTR of parental vs. persister cells was insufficient to explain the altered translation of this subset of mRNAs (Fig. 4b). Another hypothesis is that the translationally upregulated mRNAs possess or acquire a distinctive “mark” that promotes their selective increased translation. mRNA methylation, particularly m^6^A, is a posttranscriptional modification that impacts the translatome^40,41^. We thus interrogated m^6^A distribution between the 178 translationally upregulated mRNAs in persister cells and the top 180 translationally downregulated mRNAs in three different cell lines (Supplementary Data 4). Global m^6^A modifications were previously investigated by meRIP-seq in two cell lines^42,43^ (U2OS and HeLa cell lines) and by mCLIP-seq in the A549 cell line^44^. Transcriptomic view of m^6^A RNA methylations on genome-based coordinates showed that mRNAs with elevated TE in persister cells are highly methylated on their 5′-UTR and coding regions close to 5′-UTR, as compared with the 180 downregulated transcripts or total mRNAs (Fig. 4c and Supplementary Fig. 9a). Although significantly lower levels of m^6^A modifications were observed in the 3′-UTR of these 178 upregulated transcripts as compared with the 180 downregulated mRNAs, similar m^6^A modifications were observed at the stop codon site between up- and downregulated transcripts (Fig. 4c and Supplementary Fig. 9a).

m^6^A modifications convey many cues of translation control or mRNA decay^45^. The increased 5′-UTR m^6^A enrichment in the 178 mRNAs may explain their selective elevated translation levels in the context of global translational inhibition in persister cells. To explore this hypothesis, m^6^A modification of mRNAs purified from heavy polysome fractions of both persister and parental cells was studied using different strategies, including m^6^A dot blot assay, nucleoside analysis by liquid chromatography coupled to tandem mass spectrometry (LC/MS-MS) and m^6^A-seq (Fig. 4d). An enrichment in m^6^A modification in mRNAs purified from heavy polysome fractions was found in persister cells compared to parental cells (Fig. 4e “Day 1” and Supplementary Fig. 9b). In addition, we found that this m^6^A enrichment in heavy polysome fractions from persister cell was reversible, following the same dynamic of the translation remodelling associated with persistence status (Fig. 4e “Day 9”). Of note, we did not observe any differences in other mRNA-related modifications, such as N^6^, 2-*O*-dimethyladenosine (m^6^A_m_), 1-methyladenosine (m^1^A) and 7-methylguanosine (m^7^G) (Fig. 4e). To confirm the enrichment of m^6^A in heavy polysome fractions of persister cells, we performed m^6^A immunoprecipitation followed by RNA sequencing (m^6^A-seq) in both total lysates and heavy polysome fractions. We found that the 178 translationally upregulated mRNAs but not the top 180 translationally downregulated transcripts showed enrichment of m^6^A in their 5′-UTRs in both parental and persister cells (Fig. 4f, top panel). This indicates that the m^6^A modification was present before BRAFi/MEKi treament. However, there was a clear enrichment in 5′-UTR m^6^A-modified transcripts in persister cell-derived polysome fractions compared with those derived from parental cells (Fig. 4f, bottom panel), indicating that the majority of translationally upregulated transcripts in persister cell harbour m^6^A in their 5′-UTR.

To confirm whether the polysomal m^6^A enrichment is specific to the mRNAs that were upregulated at the translational level, we performed m^6^A antibody-based immunoprecipitation of mRNAs purified from heavy polysome fractions, followed by RT-qPCR (m^6^A-qPCR) with gene-specific primers. Consistent with the enrichment of m^6^A modifications and with their increased TE, the six tested transcripts selected from the translationally upregulated mRNAs were significantly enriched in m^6^A-immunoprecipitated polysomal mRNAs from persister cells as compared with parental cells (Fig. 4g and Supplementary Fig. 9d, *p* < 0.05, unpaired *t*-test). Notably, the housekeeping genes (*HPRT* and *TBP*) as well as the genes that were downregulated at the translation level (e.g., *RAPTOR* and *DUSP2*) showed no significant increase between parental and persister cells (Supplementary Fig. 9d). Similar to the m^6^A LC/MS-MS results, we observed that the m^6^A-enrichment effect on specific mRNAs was also reversible after 9 days of drug-free culture (Fig. 4g and Supplementary Fig. 9e).

### Preventing m^6^A methylation synergizes with BRAFi/MEKi

**Fig. 5:**
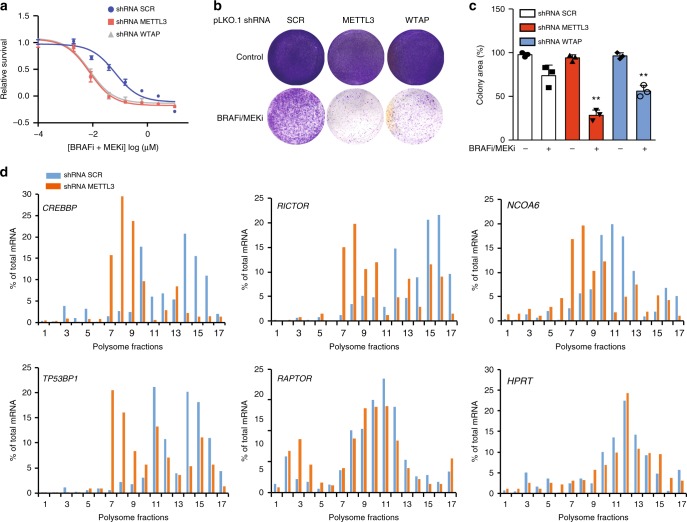
m^6^A methyltransferase knockdown inhibits the translation remodelling and the survival of melanoma persister cells. **a** Short-term WST-1 cell viability analysis of the A375 melanoma cells expressing the indicated shRNAs upon the treatment of BRAFi/MEKi for 48 h. **b**, **c** m^6^A methylatransferase shRNA knockdown abrogates BRAFi/MEKi dependent persister cell-derived colony formation. An image representation (**b**) and the quantification on the clonogenic assay of persister cells are presented (**c**) (*n* = 3, **p-value < 0.001, unpaired *t*-test). **d** RT-qPCR quantification of mRNAs in fractions obtained by sucrose-gradient ultracentrifugation of lysates from persister cells in the presence or absence of shRNA METTL3. The raw data of **a**, **c** and **d** are available in Source Data.

The RNA m^6^A modification is deposited by a methylation machinery comprising METTL3, METTL14 and WTAP^46^. Given the observed m^6^A enrichment in the 5′-UTR of the translationally upregulated mRNAs in persister cells, we hypothesized that the m^6^A methylation machinery could be involved in BRAF^V600E^ melanoma cell tolerance to BRAFi/MEKi treatment. Indeed, METTL3 or WTAP knockdown significantly increased the sensitivity to BRAFi/MEKi treatment in the BRAF^V600E^ A375 cell line (Fig. 5a and Supplementary Fig. 10a). In addition, METTL3 or WTAP knockdown significantly reduced the number of persister cell-derived colonies compared with scramble shRNA (Fig. 5b, c). This effect was accompanied by a decreased TE of some of the mRNAs that were upregulated in persister cells (Fig. 5d).

### EIF4Ai decreases polysomal m^6^A enrichment in persiter cells

**Fig. 6:**
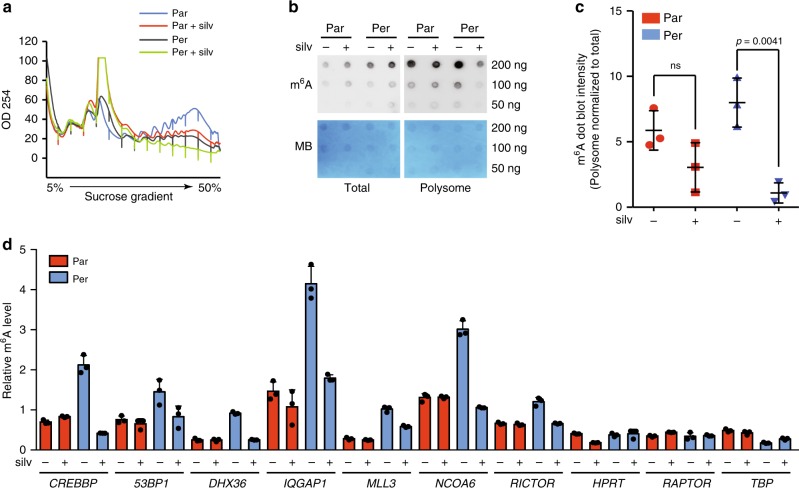
Silvestrol decreases the m^6^A enrichment in mRNAs from polysome fractions. **a** Polysome profiles of persister (Per) vs. parental (Par) cells in the presence or absence of silvestrol (silv, 30 nM for 4h). **b**, **c** Total mRNAs and polysome-bound mRNAs were purified for m^6^A dot blot analysis (**b**) and the semi-quantification of the dot blot intensity (**c**). Polysome-derived mRNA m^6^A intensity was normalized to the total lysate (input)-derived mRNA m^6^A. *n* = 3, unpaired *t*-test. **d** RT-qPCR quantification of m^6^A enrichment in the translationally upregulated mRNA in persister cells upon the indicated treatment (*n* = 3, **p-value < 0.01, unpaired *t*-test). The raw data of **c** and **d** are available in Source Data.

By interrogating a previously reported study in which an in vitro RNA pull-down assay was performed by using an RNA probe containing four repeats of the m^6^A-containing sequence^47^, we found that eIF4A1 seemed preferentially associated with the above-described RNA probe as compared with eIF4E and eIF4A3, whereas eIF4A2 was not detected (Supplementary Fig. 10b). We then investigated whether silvestrol could abrogate the polysomal m^6^A enrichment in persister cells. Although silvestrol did not decrease the level of m^6^A modification in total mRNAs, it decreased the m^6^A level in mRNAs purified from polysome fractions, with a much stronger effect in persister cells than in parental cells (Fig. 6b, c). Similarly, we observed a specific decrease in the quantities of translationally upregulated mRNAs in heavy polysome-derived m^6^A-immunoprecipitated pool by using m^6^A-qPCR, suggesting that silvestrol selectively inhibits the polysome recruitment of m^6^A-containing mRNAs in persister cells (Fig. 6d).

## Discussion

Our current understanding of mRNA translation reprogramming has been well documented in haematopoietic differentiation^48^ and heat-shock responses^49^. However, its implication in cancer persistence had not been explored. We show here that a substantial m^6^A-associated mRNA translation remodelling is correlated with the melanoma persistent state upon BRAFi/MEKi treatment. m^6^A modification in both the 5′-UTR and 3′-UTR of certain mRNAs was reported to promote a cap-independent mRNA translation upon stress responses^41,50,51^, whereas another study showed that METTL3 can promote a cap-dependent translation in a m^6^A catalytic activity-independent manner^52^. It will be interesting to further determine whether persistent state-related translationally upregulated mRNAs possess m^6^A modifications at a specific location at single-nucleotide resolution and whether m^6^A-binding complex(es) directly recruit(s) eIF4A to the subset of translationally upregulated mRNAs in melanoma persister cells.

Overall, our work describes an epitranscriptomic and reversible level of regulation in cancer persister cells. This translation remodelling appears to be an exploitable vulnerability of melanoma persister cells. The inhibition of translation remodelling by eIF4A inhibition can selectively eradicate persister cells. This represents a potential strategy by combining eIF4A inhibitors with BRAFi/MEKi to prevent resistance of BRAF^V600E^ melanoma to targeted therapies.

## Methods

### Cell lines and chemicals

The A375 and Malme-3M melanoma cell lines lines used in this study were purchased from the ATCC. The WM983B melanoma cell line was a gift from L. Larue (Institut Curie, France). The A375 and WM983B cell lines were maintained at 37 °C and 5% CO_2_ in a humidified atmosphere and grown in Dulbecco’s modified Eagle’s medium (DMEM) growth media supplemented with 10% fetal bovine serum (FBS) and 2 mM glutamine (Gibco). The Malme-3M cells were grown in Iscove’s modified Dulbecco’s medium supplemented with 20% FBS. The PC9 lung cancer cell line was grown in RPMI1640 supplemented with 10% FBS. All cell lines were regularly controlled to be mycoplasma-free by using a PCR-based test (Biovalley). BRAF inhibitor (PLX4032, #S1267), MEKi (Cobimetinib, #S8041), EGFR inhibitor (Erlotinib, #S1023), KDM6B inhibitor (GSK-J1, #S7581) and CBP/p300 inhibitor (SGC-CBP30, #S7256) were purchased from Selleckchem (Euromedex, France). Silvestrol was purchased from MedChem Tronica (# HY-13251, Sweden). Pateamine A was provided by S. Apcher (Gustave Roussy Institute, France). Hippuristanol was provided by J. Tanaka (University of the Ryukyus, Japan). 4E1RCat (#SML0197) and cycloheximide (CHX, #C104450) was purchased from Sigma. All the chemicals were dissolved in dimethylsulfoxide (DMSO) for in vitro studies. The panel of small-molecule chemical library was obtained from L. Désaubry (Strasbourg, France).

### Long-term A375 cell line viability screen

Long-term viability assays were performed by plating 5000 A375 parental cells per well (in total, 1000 wells were assayed) in duplicate 96-well plates^11^. Cells were treated in BRAFi (PLX4032, 1 μM) and MEKi (Cobimetinib, 1 μM) during 3 weeks, and fresh medium with the treatment was changed every 3 days. In the end of the treatment, cell viability was measured by WST-1 ATP-based assay (Roche, France). Absorbance values among the 1000 wells were normalized based on the *z*-score method to represent the relative cell viabilities. Wells were considered to contain persistent cells if the *z*-score value was <0.3. The three wells marked as red in Supplementary Fig. 1 were those containing proliferative melanoma cells even in the presence of BRAFi/MEKi; thus, these cells were considered as resistant cells. To be noted, the definition of persister cells is strictly linked to their reversibility in terms of the sensitivity to BRAFi/MEKi treatment.

To exclude the possibility of pre-existing resistant clones in the parental cells, we generated single cell-derived sub-clones and repeated the long-term viability assay in each clone. Briefly, A375 parental cells were seeded into 96-well plates at a density of 0.25 cells/well. After 2 weeks, ~15% of wells contained colonies of about ~10,000 cells. A minority of wells contained two colonies, which were easily distinguishable. Wells containing only a single colony were expanded an additional ~10 doublings for use in experiments. Therefore, we tested five single cell-derived sub-clones (A375E1–E5) for the long-term viability assay.

### Generation of drug-tolerant persister cells

For the melanoma cell lines, parental cells were treated with PLX4032 (1 μM) and Cobimetinib (1 μM) for 72 h; ~80% of cells were killed and detached by the combination treatment. Survival attached cells were trypsinized and washed once in phosphate-buffered saline (PBS). These recovered persister cells were re-cultured in drug-free medium for a period of 9 days and experiments were performed during this period on Day 1, 3, 6 and 9 (Fig. 1b). Parental cells treated with DMSO were used as control.

### WST-1 cell viability assay

Cell sensitivity to the treatments was measured using WST-1 reagent (Roche Applied Science). Parental and drug-tolerant persister cells were plated in 96-well plates (5000 cells/well). After 24 h, the cells were treated with drugs or DMSO at the indicated concentrations, in triplicate. WST-1 reagent was added to each well (5 μL per 100 μL of medium) and incubated at 37 °C for 2 h before and after the treatment period of 48 h. The plates were then read at 450 nm on a Victor Multi-label Counter model 1420 (Perkin Elmer). Cell sensitivity is represented as percentage of the absorbance compared with mock-treated cells.

### In vitro generation of resistant cell lines

We used a single cell-derived sub-clone (A375E2; Supplementary Fig. 1a) to generate resistant cell lines. This clonal parental cell line offered a unified basis to identify and interpret genetic changes. Parental A375E2 cell line was treated with a high dose of PLX4032 (1 μM) and cobimetinib (1 μM) for 72 h. Drug-tolerant persister cells were recovered by trypsinization and were equally split into ten 60 mm petri dishes. After 24 h, all the persistent cells were treated with escalating concentration of PLX4032 and cobimetinib for over 3 months. The starting concentrations of PLX4032 and cobimetinib were determined from their IC50, then the concentrations of the combination treatment were increased five times of the precedents if the cells start to proliferate. The augmenting concentration of the treatment was stopped when the concentration of PLX4032 was arrived at 1 μM and the concentration of cobimetinib was arrived at 0.5 μM (Supplementary Fig. 2b). The resistant cells were then maintained in culture with 1 μM PLX4032 and 0.5 μM cobimetinib. To determine the obtained clonal cell lines are real acquired resistant cells, we withdrew the combination treatment and cultured them in drug-free medium for ~10 passages (~1 month of culture). The cells were then subjected to the re-challenge of PLX4032 and cobimetinib for 48 h and cell sensitivity was measured using WST-1 assay. To further confirm their resistance is irreversible, we performed clonogenic assay on all the resistant clones after ~10 passages of drug-free culture.

### Clonogenic assay

For the single cell-derived resistant clones, cells were cultured in drug-free medium for ~10 passages and then were plated at a low density (5000 cells per well in 6-well plates) in fresh medium. After 24 h, cells were treated with drugs or DMSO at the indicated concentrations, in duplicate. After 2 weeks, cells were washed once in PBS and fixed with 4% paraformaldehyde for 12 min at room temperature. Cells were then stained with 0.5% (w/v) crystal violet in 70% ethanol.

For persister cell clonogenic assay in different drug combination schedules, we generated the A375 persister cells upon PLX4032 (1 μM) and cobimetinib (1 μM) for 72 h. The residual persister cells were then plated at a low density in six-well plates. Cells were then treated with PLX4032, cobimetinib and silvestrol in different combination schedules as indicated in Fig. 3h. for 6 weeks. Control cells were left in DMSO containing DMEM medium supplemented with 10% FBS. After 6 weeks, cells were washed once with cold PBS and fixed with 4% paraformaldehyde for 12 min at room temperature. Cells were then stained with 0.5% (w/v) crystal violet in 70% ethanol. Images were analysed by ColonyArea^53^.

### Whole-exome sequencing and analysis

Genomic DNA was extracted from confluent 100 mm plates using PureLink genomic DNA extraction kit (Invitrogen, #18200) according to the manufacturer’s procedure. A single band of DNA that was >20 kb with no degradation was detectable using agarose gel electrophoresis. The Genomic DNA (1 μg in 50 μL water) was submitted to NGS genomic platform of Institut Curie (Paris, France) for quality control, library preparation and WES. Sequencing was performed at Institut Curie on an Illumina HiSeq 2000 sequencer with 30× coverage per sample.

Data were processed and aligned to the reference genome hg19 using Burrows-Wheeler alignment tool, BWA ALN. Copy number variations were called using the Sequenza with default settings^54^. The algorithm was applied for the WES data from resistant clones (DR clones) compared with parental single cell-derived sub-clone (A375E2) as matched normal sample. The allele-specific copy number was estimated by the average depth ratio and B allele frequency for each segment using a probabilistic model. Somatic single-nucleotide variants (compared with parental A375E2 sub-clone) were called using MuTect^55^ with default parameters. Significant single-nucleotide variants that are called in either COSMIC database (http://cancer.sanger.ac.uk/cosmic) and/or SNP database were plotted as heatmap.

### m^6^A-seq

A375 cells were subjected to polysome profiling and the last four heavy polysome fractions were collected. Polysome fraction and total lysate were then subjected to RNA extraction by using TRIzol reagent (Sigma). RNA integrity was confirmed by Bioanalyzer RNA Nano chips (Agilent Technologies). PolyA+ RNA was prepared using one round of the GenElute mRNA prep kit (Sigma) by using 60 μg total RNA with ~5% residual rRNA as confirmed by Bioanalyzer RNA Nano chips. RNA was fragmented using fragmentation reagent (Life Technologies). mRNA fragments were precipitated with ethanol and were used for m^6^A immunoprecipitation (m^6^A antibody, Synaptic Systems), or IgG control was performed in precipitation buffer (50 mM Tris, pH 7.4, 100 mM NaCl, 0.05% NP-40, 1 mL total volume) with 1 μL RNasin Plus (Promega) rotating head over tail at 4 °C for 4 h, followed by incubation with 30 μL magnetic Protein A/G beads (Thermo Fisher Sicentific) rotating at 4 °C for 2 h. Bead-bound antibody-RNA complexes were recovered and were washed twice with immunoprecipitation buffer. Fragments were eluted by Proteinase K treatment. RNA was recovered from the eluate using TRIzol LS following the manufacturer’s recommendations. Sequencing libraries were prepared using the Illumina TruSeq stranded mRNA protocol following the standard procedure starting from mRNA fragments recovered from m^6^A-IP, IgG-IP and 100 ng of Poly-RNA input fragments. Libraries were quality-checked using Bioanalyzer DNA chips (Agilent Technologies) and sequencing was performed on Illumina HiSeq4000 PE 2x100 multiplexing all samples per experiment. The entire procedure was performed in LC Science, Co. (Huston, TX, USA).

### Western blotting analysis

Western blotting was performed on cell extracts from both parental and persister cells treated with indicated concentrations of drugs. Immunoblots were performed from whole cell lysate prepared using RIPA buffer (Cell Signaling Technology) supplemented with dithiothreitol (DTT), phenylmethylsulphonyl fluoride (Sigma), and protease and phosphatase inhibitors (Roche). Cell lysates were quantified for protein content using a bicinchoninic acid protein assay kit (Invitrogen). Protein samples were resolved on NuPAGE 4–12% Bis-Tris gels with MOPS buffer or 3–8% Tris-acetate gels with Tris-acetate buffer (Life Technologies) and then transferred to 0.45 μm nitrocellulose membrane (Amersham). After saturation in Tris-buffered saline buffer supplemented with 5% powdered milk, the membranes were incubated with antibodies (diluted at 1:1000 if not indicated) overnight at 4 °C with agitations. CBP (Cell Signaling Technology, rabbit, #7389), TP53BP1 (Bethyl lab, rabbit, #A300-272A), MLL3 (Novus, rabbit, #45880002, 1:100 dilution), phospho-S6 (Cell Signaling Technology, rabbit, #4858), phospho-Akt (Cell Signaling Technology, rabbit, #9271), Akt (Cell Signaling Technology, rabbit, #9272), phospho-ERK1/2 (Cell Signaling Technology, rabbit, #9101), ERK1/2 (Cell Signaling Technology, rabbit, #4695), GAPDH (Sigma, #SAB2100894), BRAF (Novus, #NBP1-47668), phospho-4EBP1 (Cell Signaling Technology, rabbit, #9451), 4EBP1 (Cell Signaling Technology, rabbit, #9452), and H3K9Ac (Cell Signaling Technology, rabbit, #9649) were used. Horseradish peroxidase (HRP)-conjugated secondary antibodies were purchased from Sigma.

### BRAF alternative splicing PCR analysis

A375E2 cell line and A375DR clones were subjected to total RNA extraction using TRIzol-chloroform according to manufacturer’s instruction. The quality of RNA was controlled using Agilent RNA 2100 Bioanalyzer. Two hundred nanograms of RNAs were reverse transcribed using SuperScript IV reverse transcriptase (Invitrogen). BRAF^V600E^ double kinase domain splicing was then verified using forward primer targeting exon 18 and reverse primer targeting exon 10^56,57^. Forward primer: 5′-ATTCTCGCCTCTATTGAGCT-3′; Reverse primer: 5′-AAGGCTTTCACGTTAGTTAG-3′.

### Polysome profiling and exon-array experiment

Polysome profiling was performed based on previous report^9^. Parental and persister cells with indicated treatment were incubated at 37 °C with 100 μg/mL cycloheximide in fresh medium for 5 min. Cells were then washed, scraped into ice-cold PBS supplemented with 100 μg/mL cycloheximide and centrifuged at 3000 r.p.m. for 5 min. The cell pellets were re-suspended into 400 μL of LSB buffer (20 mM Tris, pH 7.4, 100 mM NaCl, 3 mM MgCl_2_, 0.5 M sucrose, 1 mM DTT, 100 U/mL RNasin and 100 μg/mL cylcoheximide). After homogenization, 400 μL LSB buffer supplemented with 0.2 % Triton X-100 and 0.25 M sucrose was added. Samples were stayed on ice for 30 min and centrifuged at 12,000 × *g* for 15 min at 4 °C. The supernatant was adjusted to 5 M NaCl and 1 M MgCl_2_. The lysates were then loaded onto a 5–50% sucrose density gradient and centrifuged in an SW41 Ti rotor (Beckman) at 36,000 r.p.m. for 2 h at 4 °C. Polysome fractions were monitored and collected using a gradient fractionation system (Isco). Polysome-bound RNAs were extracted using TRIzol (Sigma) according to manufacturer’s procedure and were quantified by using RNA 2100 Bioanalyzer (Agilent Genomics). Exon array experiments were submitted to NGS platform (Institut Curie) and performed in triplicate using Affymetrix Clariom D human array (Affymetrix). For transcriptomic analysis, total RNAs were extracted using Trizol (Sigma) and quantified by using 2100 Bioanalyzer (Agilent Genomics). Exon arrays were performed on total RNAs in triplicate.

### Genome-wide transcriptome and translatome analysis

Exon array raw data CEL files were processed with Affymetrix expression console software. Data were then normalized based on SST-RMA method using default settings. Principal component analysis on each replicate samples was performed to interrogate the reproducibility of the replicates (Supplementary Fig. 4c). Gene expression counts based on exon alignment were used for statistical modelling of the polysome profiling data using R software. The Negative Binomial (NB) model was well fitted to the gene expression data (Supplementary fig. 4d). NB model has been widely used to estimate the distributions of gene expression counts across samples, and compared to Poisson model, NB is much more flexible, and it allows technical or biological variability that might lead to a variance higher than the mean^58^. The TE was calculated using Xtail software^59^. Briefly, for each gene, normalized read counts of total mRNAs or polysome-bound mRNAs in all samples were used to fit NB distribution with dispersions α and means μ. mRNA count K for gene *j* in sample *i* is described as *K*_*ji*_ ~ NB(*μ*_*ji*_, *α*_*ji*_). The raw gene expression data were then scaled by a normalization factor using the median-of-ratios normalization method by using DESeq2 and the posterior mean and dispersion of both total mRNA and polysome mRNA were estimated separately for each gene using empirical Bayes shrinkage. Xtail then defines the translational variation across two conditions as the difference between the log2FoldChange (log2FC) of polysome mRNA and total mRNA, or between the log2 ratios of polysome mRNA to total mRNA (log2R). The software establishes a probability distribution for the translational changes, which was used to infer statistical significance of differential translations. Posterior probability for a given coefficient β_j_ was calculated by log Pr(*β*_*j*_) = ${\sum} {{\mathrm{log}}\,{\mathrm{f}}_{{\mathrm{NB}}}{\mathrm{(K}}_{{\mathrm{ji}}}{\mathrm{,m}}_{{\mathrm{ji}}}{\mathrm{(b}}_{{\mathrm{ji}}}{\mathrm{),a}}_{{\mathrm{ji}}}{\mathrm{)}}}$. Finally, Xtail tests for each gene whether there is a significant difference between log2FC of polysome mRNA and total mRNA in two conditions. Genes with log2FC more than 1 (*p* < 0.05) were defined as significant genes, which allowed us to define groups of genes that were regulated only at translation level or only at transcription level (Fig. 2a). The *p*-values were further calculated by sample permutations (parental vs. persister); sample permutation results were shown in Supplementary Fig. 4e. Genes that were upregulated at the translation level were further studied in the following experiments.

### 5′-UTR sequence analysis

Genes that were downregulated (top 180 genes) and upregulated (178 genes) at the translational level in persister cells were selected. Their 5′-UTRs were retrieved from UCSC genome browser. The free energy of RNA folding, length of the 5′-UTR and the GC content of the corresponding 5′-UTR were analysed in mfold Web Server (http://unafold.rna.albany.edu) with the default parameters. Among all of the 5′-UTR sequences analysed, the lowest free energy was selected to compare between the two gene sets.

### Polysome fraction quantitative PCR analysis

For RT-qPCR experiments, RNA was extracted by TRIzol-chloroform method from 250 μL of each fraction, comprising both monosome fractions and polysome fractions. Extracted RNA from each fraction was diluted in 30 μL RNase-free water. Same volume of RNA from each fraction was used to prepare cDNA by using SuperScript IV Reverse Transcriptase (Invitrogen) with random hexamer primers according to the manufacturer’s instructions. Following the reverse transcription, mRNA abundance was determined by qPCR using Luminaris SYBR green PCR Master mix (Thermo Scientific) with the indicated primers. Data were analysed by the threshold cycle (Ct) comparative method and quantified as percentage of the total RNA considering the whole fractions stand for 100%. *HPRT* gene was used as a control. PCR primers are listed below:

*CREBBP* forward 5′-TCAGTCAACATCTCCTTCGC-3′

reverse 5′-TGTTGAACATGAGCCAGACG-3′

*MLL3* forward 5′-GGGCTGGAGACAACAGAAAC-3′

reverse 5′-CAACCAGACTGAGTTCATCCC-3′

*NCOA6* forward 5′-TCCTCTCTGGGCTCCATATAC-3′

reverse 5′-GCTGGGTTCATTTGTCTGTTC-3′

*TP53BP1* forward 5′-GCTGGAGAAGAACGAGGAGACG-3′

reverse 5′-CCTTACTGGGCTGTGCTGTC-3′

*DHX36* forward 5′-CATGGATGAACGACGAGAAGAAC-3′

reverse 5′-CCACAACCAGTTTCACCAC-3′

*ARID5B* forward 5′-CTGTCCATTCCTTCCCAAGGC-3′

reverse 5′-GCAATCCATTCAAGCCAACAAG-3′

*RICTOR* forward 5′-CAACTGGGATGCTGTGAGGCATAG-3′

reverse 5′-GTACTAGTAGAGCTGCTGCCAAAC-3′

*RAPTOR* forward 5′-GAGAAGCTCTACAGCCTCCTCTCC-3′

reverse 5′-CCGTCCTCTCTGCAGAGTTGCC-3′

*IQGAP1* forward 5′-TCCATTACTTAGGAAAGAGTGGAAACT-3′

reverse 5′-CAAACACCAAAGCTTACAATATAGTACTGC-3′

*HPRT* forward 5′-GCTGAGGATTTGGAAAGGGTGT-3′

reverse 5′-CCATCTCCTTCATCACATCTCG-3′.

*TBP* forward 5′-CACCTTATGCTCAGGGCTTGG3′

reverse 5′-GTGGAGTAAGTCCTGTGCCG-3′.

*DUSP2* forward 5′-CCACTGCCGTGTACTTCCTG-3′

reverse 5′-GTTGAGGACGGCTGTGATGC-3′.

### Lentiviral shRNA stable cell lines

The pLKO.1 vector-based shRNA lentiviral constructs were purchased from Sigma TRC Mission shRNA library. All the shRNAs were chosen based on experimentally validated datasheet (Sigma). Lentivirus was packaged by co-transfection of constructs with the third generation packaging plasmids pMD2.G, pRRE and pRSV/REV with Calfectin (Thermo Fischer Scientific) into 10 cm plates HEK293T cells. Medium was changed after 24 h, the 48 h and 72 h supernatants were collected, centrifuged at 3000 r.p.m. for 10 min and filtered through a 0.45 μm filter. The lentivirus particles were then aliquoted and stored at −80 °C. A375 cells were transduced with shRNA lentivirus particles and stable cell lines (shSCR, shMETTL3, shWTAP) expressing each individual shRNA were selected with 1 μg/mL puromycin. Sensitivity to BRAFi/MEKi treatment was tested by using WST-1 viability assay.

### m^6^A public dataset analysis

To analyse the m^6^A modification in the 178 genes upregulated at translation level in persister cells, we interrogated three published m^6^A genome-wide dataset. One dataset was from A549, a human non-small cell lung cancer cell line whose m^6^A modification was analysed by mCLIP-seq, another two data sets were obtained from two different human cell lines, HeLa and U2OS, whose m^6^A modification was analysed by MeRIP-seq. The m^6^A modification of the corresponding 178 genes and the top 180 genes that were downregulated at the translation level in melanoma persister cells were extracted manually from the three dataset and were registered as BED format files, respectively. The m^6^A distribution of the two populations of transcripts was plotted by using Guitar R^60^. The m^6^A modification of total RNAs was plotted as a control.

### m^6^A level dot blot

Total RNA or heavy polysome-bound RNA was isolated according to the manufacturer’s instructions using TRIzol reagent (Sigma). The RNA was re-suspended in ultrapure RNase-free water and subjected to RNA clean up reaction with RNeasy Midi kit (Qiagen), according to the manufacturer’s protocol. RNA was eluted in ultrapure RNase-free water. PolyA RNA was purified using Dynabeads mRNA purification kit (Life Technologies) according to the manufacturer’s instructions. Purified RNA was re-suspended in ultrapure RNase-free water and quantified with NanoDrop 2000 (Thermo Fischer Scientific). RNA was then prepared at three concentrations by serial dilution, and was denatured at 95 °C to disrupt secondary structures in a heat block for 2 min. RNA was then chilled on ice immediately after denaturation. Two microlitres of each concentration of RNA was loaded manually onto Amersham Hybond-XL (#RPN303s) membrane. The membrane was then cross-linked with Stratalinker UV Cross-linker twice at 1200 microJoules (×100) for 25 s each time. Membrane was then washed in 10 mL PBS buffer containing 0.02% Tween-20 (Thermo Fischer Scientific) for 10 min at room temperature, followed by incubation in 10 mL PBS buffer containing 0.02% Tween-20 and 5% milk for 1 h at room temperature with gentle shaking. The membrane was then washed once with PBS buffer containing 0.02% Tween-20 and was incubated with anti-m^6^A antibody (1:1000 dilution, Active Motif, #61755) in PBS buffer containing 0.02% Tween-20 and 5% milk for overnight at 4 °C with gentle agitation. The membrane was then washed three time for 5 min each in 10 mL PBS buffer containing 0.02% Tween-20 at room temperature, followed by 1 h incubation with goat anti-rabbit IgG-HRP (1:10,000 dilution, Sigma) in 10 mL PBS buffer containing 0.02% Tween-20. The membrane was then developed with ECL western blotting substrate (Bio-rad). Dot blot was quantified by Image J and the polysome fraction-derived m^6^A level was normalized to total lysate (input)-derived m^6^A level.

### Nucleoside modification analysis by LC-MS/MS

Two hundred nanograms of PolyA+ RNA were decapped with 5 U of RppH (New England Biolabs) for 2 h at 37 °C. Decapped mRNA were subsequently digested to single nucleotides with 1 U of Nuclease P1 (Sigma) for 2 h at 42 °C. Nucleotides were then dephosphorylated into nucleosides with 1 U of Alkaline phosphatase for 2 h at 37 °C. The sample were filtered (0.22 μm pore size, 4 mm diameter, Millipore) and 10 μL of the solution was analysed by LC-MS/MS. The nucleosides were separated using an Agilent 1290 LC systems (Agilent Technologies) on a Synergi^TM^ Fusion-RP column (250 × 2.1 mm, 4 µm particle size, 80 Å) (Phenomenex, 00G-4424-B0) at a flow rate of 400 µL/min and a temperature at 35 °C. A 30 min multi-step gradient was performed using 5 mM ammonium acetate adjusted to pH 5.3 with acetic acid (solvent A) and pure acetonitrile (solvent B). Solvent B start at 0% and increase to 8% at 13 min and to 40% at 23 min with a linear gradient. The column was flushed during 2 min and then re-conditioned with 0% for 4.5 min. Detection of nucleosides was performed on an Agilent TripleQuad 6490 (Agilent Technologies) in positive electrospray ionization (ESI) mode with MassHunter Acquisition version B.06. The multiple reaction monitoring (MRM) transitions used for detection were : m/z 282–150 for m^6^A (Fragmentor voltage: 380 V; Collision energy: 21 V; Cell accelerator voltage: 1.5 V; Retention time: 16.5 min), m/z 268–136 for A (Fragmentor voltage: 380 V; Collision energy: 18 V; Cell accelerator voltage: 1 V; Retention time: 13.1 min), m/z 296–150 for m^6^Am (Fragmentor voltage: 380 V; Collision energy:18 V; Cell accelerator voltage: 1 V; Retention time: 17.6 min) and m/z 282–136 for Am (Fragmentor voltage: 380 V; Collision energy: 14 V; Cell accelerator voltage: 1 V; Retention time: 15.6 min). The ESI source was set as follows: capillary tension 2000 V, nebulizer 50 psi, gas flow rate 15 L/min, gas temperature 290 °C, sheath gas flow rate 12 L/min, sheath gas temperature 400 °C. The MS was operated in dynamic MRM mode with a retention time window of 3 min and a maximum cycle time set at 800 ms. The peak areas were determined using Skyline 4.1 Software (University of Washington, Seattle, WA, USA) and the ratios m^6^Am/A, Am/A and m^6^A/A were calculated.

### m^6^A RNA immunoprecipitation

Immunoprecipitation of m^6^A was adapted from the protocol of EpiMark N6-methyladenosine Enrichment kit (New England Biolabs). Heavy polysome-bound RNA was isolated by using TRIzol reagent (Sigma) as mentioned above and enriched for mRNA using Dynabeads mRNA purification kit (Thermo Fischer Scientific) following the manufacturer’s instruction. The isolated mRNA was re-suspended in ultrapure RNase-free water and quantified with NanoDrop 2000 (Thermo Fischer Scientific). To bind antibody to the beads, the protein G magnetic beads (Invitrogen) were pre-incubated with 1 μg of anti-m^6^A antibody (Active Motif #61755) in IP buffer (150 mM NaCl, 10 mM Tris-HCl pH 7.5, 0.1% NP-40) at 4 °C overnight. Subsequently, the beads were washed twice in IP buffer and incubated with purified mRNA for 4 h with head-to-tail rotation at 4 °C and 10% of the RNA material was kept as input control. The beads were then washed three times in high-salt wash buffer (500 mM NaCl, 10 mM Tris-HCl pH 7.5, 0.1% NP-40, 0.05% SDS). RNA was then extracted with TRIzol (Sigma) following the manufacturer’s instruction. For quantification of m^6^A enrichment, both input and immunoprecipitated RNA samples were examined by RT-qPCR as described above.

### Reporting summary

Further information on research design is available in the Nature Research Reporting Summary linked to this article.

## Supplementary information

Supplementary Information

Peer Review

Reporting Summary

Description of Additional Supplementary Files

Supplementary Data 1

Supplementary Data 2

Supplementary Data 3

Supplementary Data 4

## Data Availability

A reporting summary for this Article is available as a Supplementary Information file. The Clariom D exon-array and m^6^A-seq data sets generated for this study are available from the NCBI GEO database under accession number GSE137726. The whole-exome sequencing data sets generated from A375 resistant clones are available from the Sequence Read Archive (SRA) under accession number PRJNA573468. m^6^A-seq public data sets used in this study are acquired from GSE48037 for U2OS cell line, GSE46705 for HeLa cell line and GSE76367 for A549 cell line. The source data underlying Fig. 1b, Fig. 2d, e, f, g, Fig. 3b, c, e, f, g, i, Fig. 4e, g, Fig. 5a, c, d and Supplementary Fig. 1c, d, Supplementary Fig. 2b, Supplementary Fig. 3a, Supplementary Fig. 7a, c, d, Supplementary Fig. 8b, c, d, e, f and Supplementary Fig. 10a are provided as a Source Data file. All data are available from the corresponding author upon reasonable request.

## Source data

Source Data
