# Supplementary material for: An epitranscriptomic mechanism underlies selective mRNA translation remodelling in melanoma persister cells
Source: Nat Commun. 2019 Dec 16;10:5713. doi: 10.1038/s41467-019-13360-6 (PMC6915789; doi:10.1038/s41467-019-13360-6)
Supplement: Supplementary file 1 — Supplementary Information [file 41467_2019_13360_MOESM1_ESM.pdf]

Supplementary Information

An epitranscriptomic mechanism underlies selective mRNA translation remodelling in melanoma persister cells

Shen et al.

Supplementary Figure1-10

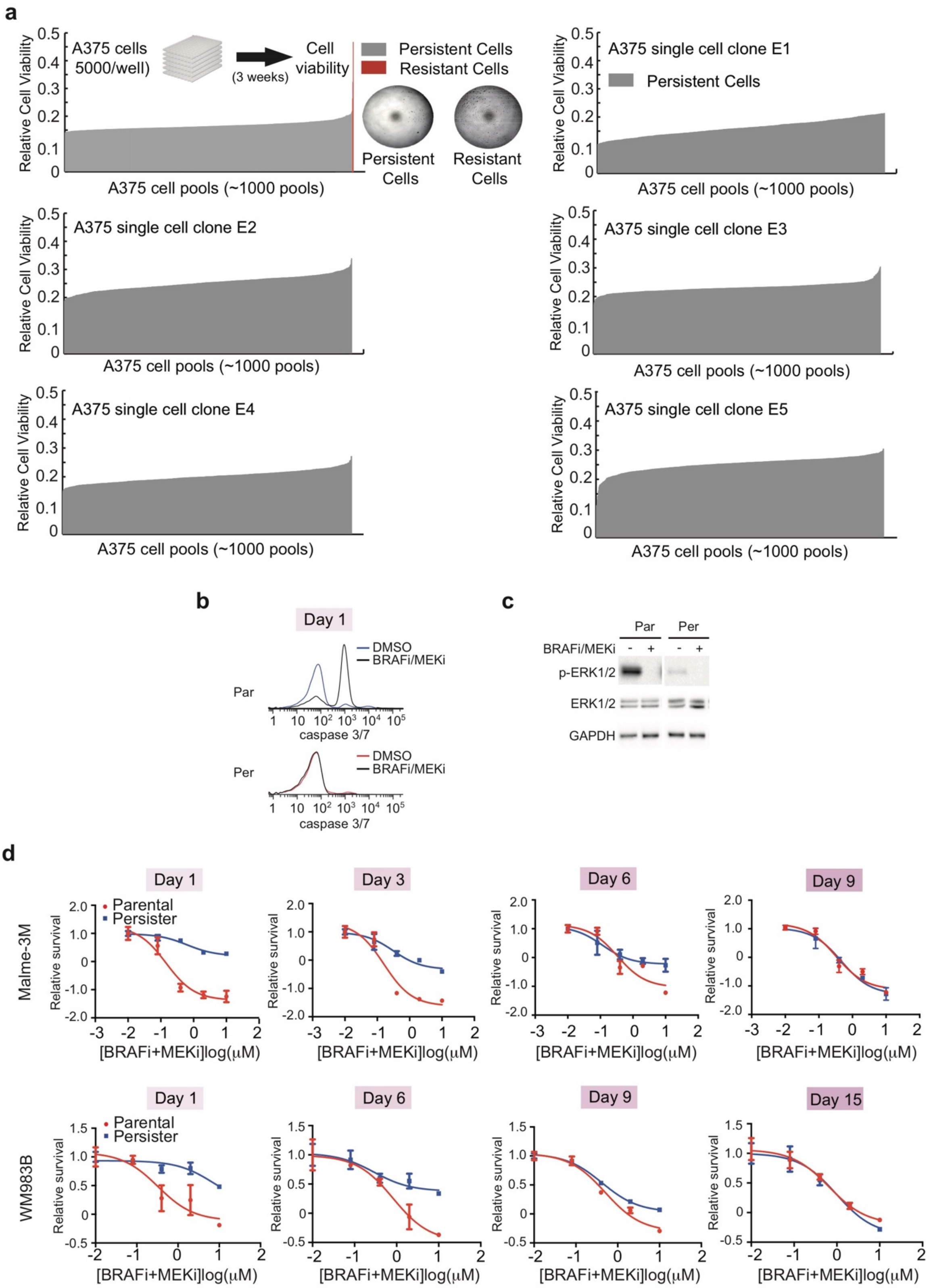

Supplementary Figure 1. Persistent state represents a major survival mechanism against BRAFi/MEKi treatment in melanoma.

(a) A375 parental cells were plated into 1000 wells (pools) in 96-well plates and treated with BRAFi (PLX4032, 1 μM) and MEKi (Cobimetinib, 1 μM) for 3 weeks. The relative viability of cells was evaluated using WST-1 assay. Each column represents one well (pool) of cells. The wells in which resistant cells start to proliferate (i.e. resistant cells) are marked as red (relative viability > 0.3)[3], the wells in which rare tolerant cells survive the treatment (i.e., persister cells) are marked as grey. Representative images are shown for persistent and resistant cells. A375 parental cells were then sub-cloned into single cell-derived clones (E1-E5), and the same viability experiments were performed on each clone. (b) Flow cytometry analysis of cell death. A375 parental and persister cells were re-challenged with BRAFi (PLX4032, 1 μM) and MEKi (Cobimetinib, 1 μM) for 48 h, and cells were stained with a caspase 3/7 fluorescent probes (Thermo Fischer Scientific, #10423) and analysed by flow cytometry. DMSO was used as a control for the treatment. (c) Western blot analysis of MAPK pathway activity in parental and persister cells. (d) Two $BRAF^{V600E}$ mutated melanoma cell lines, WM983B and Malme-3M, were treated with BRAFi (PLX4032, 1 μM) and MEKi (Cobimetinib, 1 μM) for 72 h to generate the drug tolerant persister cells. Tolerant cells were cultured in drug-free medium during a period of ~2 weeks, and then re-challenged with PLX4032 and Cobimetinib on the indicated days. The raw data of Supplementary Figure 1c and 1d are available in Source Data.

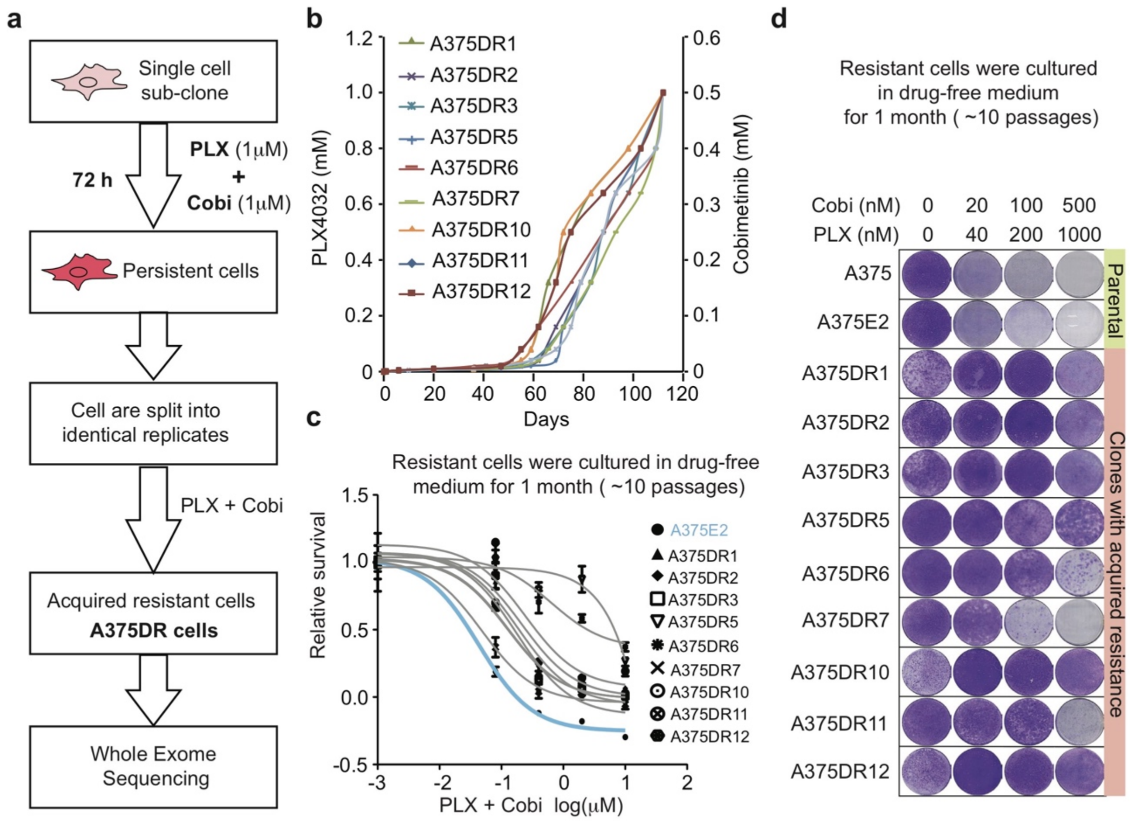

Supplementary Figure 2. Generation of melanoma resistant clones via persister cells.

(a) Schematic procedure of the generation of double resistant clones from single cell sub-clone-derived persister cells. (b) Development of A375DR cell lines after culturing single cell sub-clone-derived persister cells in the presence of BRAFi (PLX4032) and MEKi (Cobimetinib), drug concentrations were increased when the cells started to grow in the previous concentrations. (c, d) Irreversibility of the resistant state of A375DR cell lines. A375DR cell lines were cultured in drug-free medium for 10 passages. The cells were then subjected to sensitivity analysis (c) and long-term clonogenic assay upon re-challenge with BRAFi and MEKi (d). The raw data of Supplementary Figure 2b are available in Source Data.

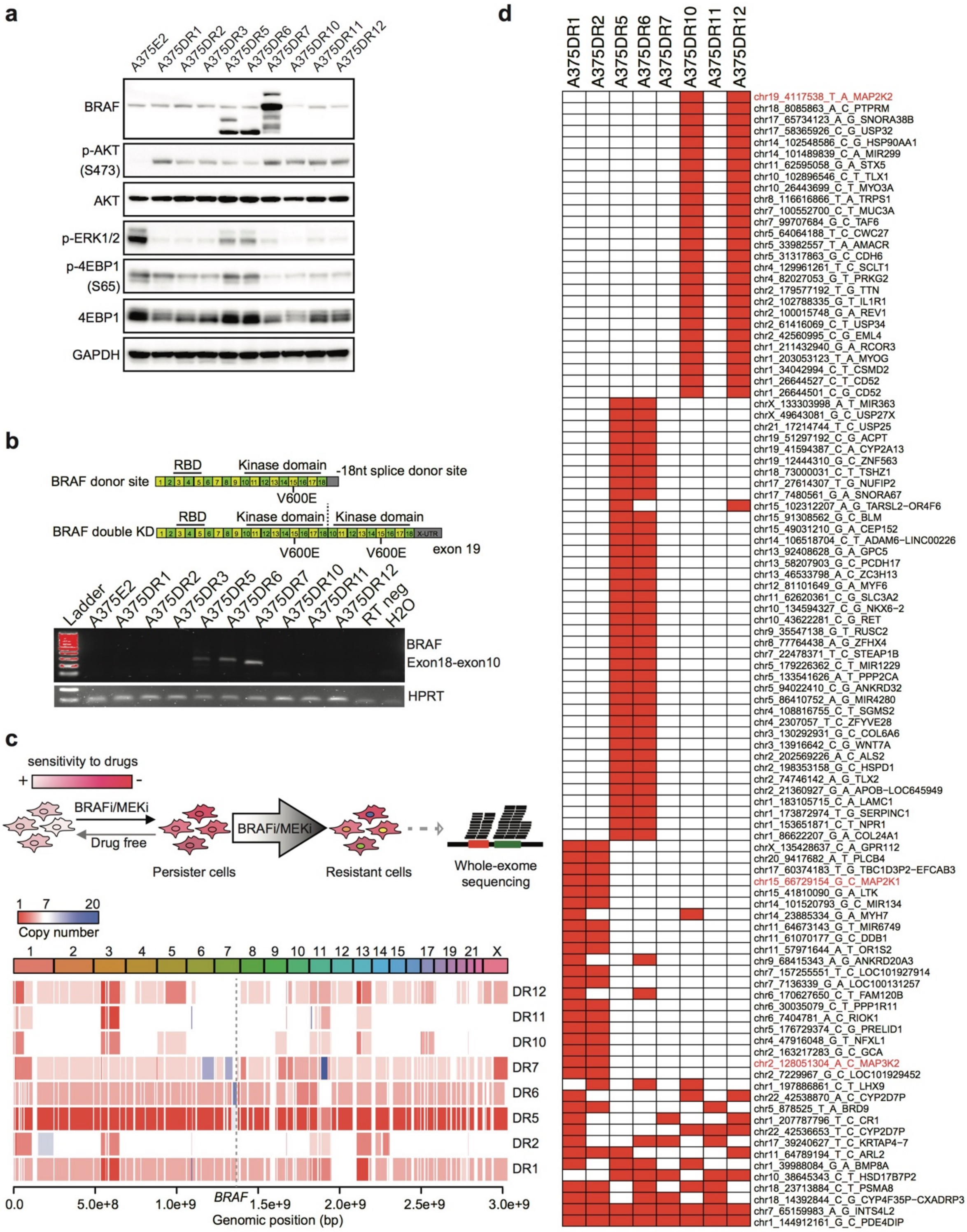

Supplementary Figure 3. Characterization of persister cell-derived melanoma resistant clones. (a) Immunoblotting detection of BRAF alternative splicing products, and activities of MAPK- and PI3K/AKT-related pathways in A375DR clones and A375E2 parental cell line. (b) PCR analysis of BRAF alternative splicing. Top panel: illustration of $BRAF^{V600E}$ and $BRAF^{V600E}$

double kinase domain encoding mRNA. Upper row indicates $BRAF^{V600E}$ with normal 3'-UTR, lower row indicates $BRAF^{V600E}$ double kinase domain splicing. Dashed line indicates splice donor site localized within exon 18, which can be used for alternative 3'-UTR splicing. Lower Panel: PCR product using a forward primer in exon 18 and a reverse primer in exon 10 validates the presence of $BRAF^{V600E}$ double kinase domains in the A375DR7 clone. (c) Genetic evolution of diverse mutant resistant clones from single-cell, subclone-derived persister cells upon continued exposure to BRAFi and MEKi. The genomic DNA of A375 DR clones was subjected to whole-exome sequencing. Top panel: illustration of the experiment; bottom panel: copy number profiles of DR clones that were derived from single-cell, subclone-based persister cells. Column: chromosome numbers and corresponding genomic positions; row: A375 DR clones. (d) Comparison of genetic alterations in A375DR clones (columns) for genes that were identified as novel mutations compared to the A375E2 parental cell line. Genetic alterations were detected using MuTect software, and genes (rows) with genetic alterations that were annotated in COSMIC and/or SNPdb are presented. Red indicates the presence of a non-synonymous single nucleotide variant versus A375E2; White indicates the absence of the corresponding mutation. The raw data of Supplementary Figure 3a are available in Source Data.

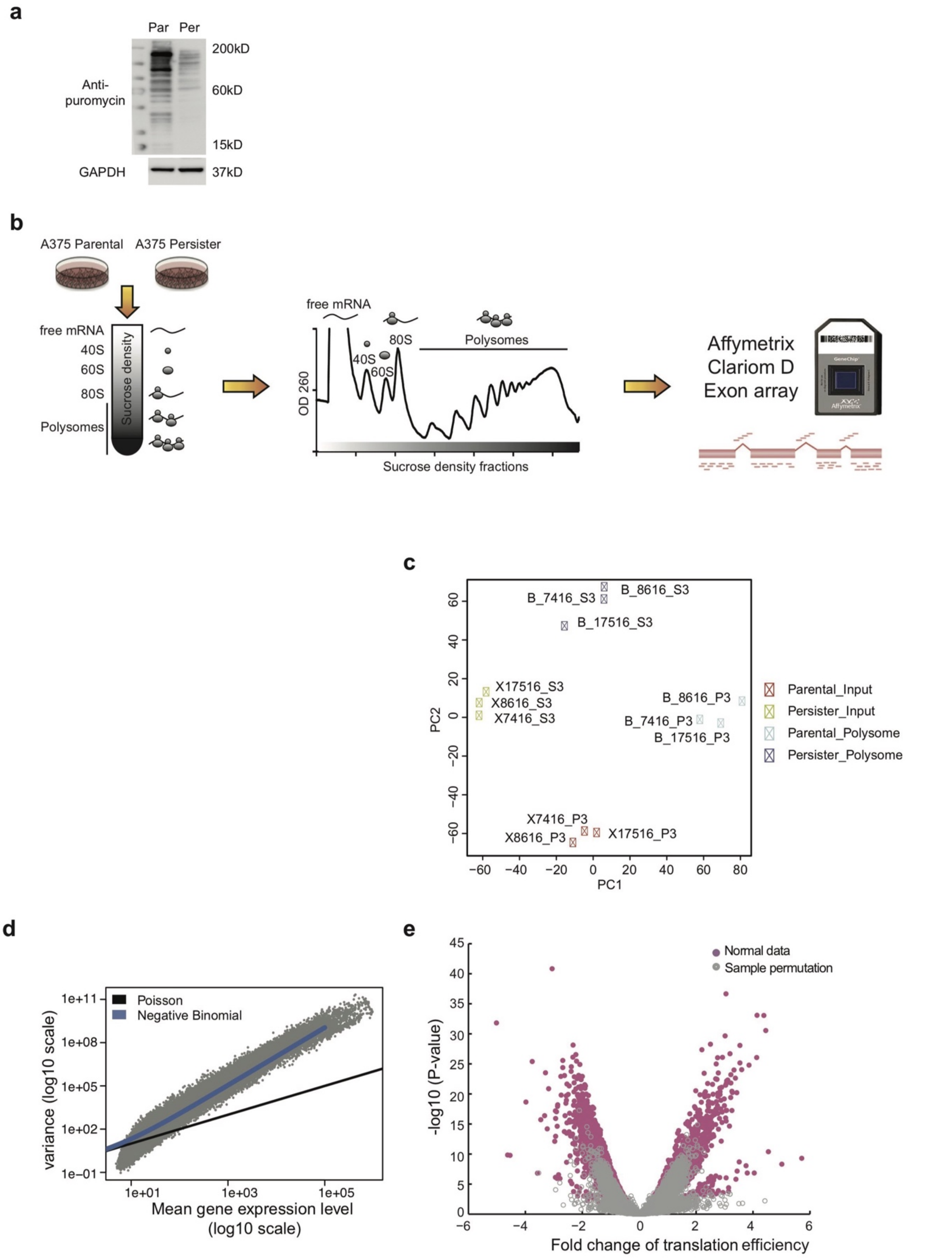

Supplementary Figure 4. Exon array-based polysome profiling analysis. (a) A375 parental and persister cells were treated with puromycin (1μg/mL) for 30 min and then harvested for protein extraction. Western blot analysis was performed with anti-puromycin to assess the basal level

of global translation. (b) RNAs from A375 parental and persistent cells were fractionated in 5 ~ 50 % sucrose density gradient. Polysome-bound RNAs were extracted based on their A260 absorbance profile and subjected to Exon array analysis in triplicates. (c) Principal component analysis showed good reproducibility among the triplicates and revealed that global expression patterns differed between the conditions. (d) Statistical modeling of the normalized data from all the conditions showed that Exon array-based gene counts followed a Negative-Binomial (NB) distribution rather than a Poisson distribution. This statistical model is the foundation of the Bayes Estimation in the translation efficiency analysis. (e) Parental and persister-derived RNA samples were permutated and the translation efficiency analysis was re-performed using the same settings in Xtail software. Sample permutations significantly decreased the differential translational regulation of mRNAs, consolidating the translation efficiency analysis.

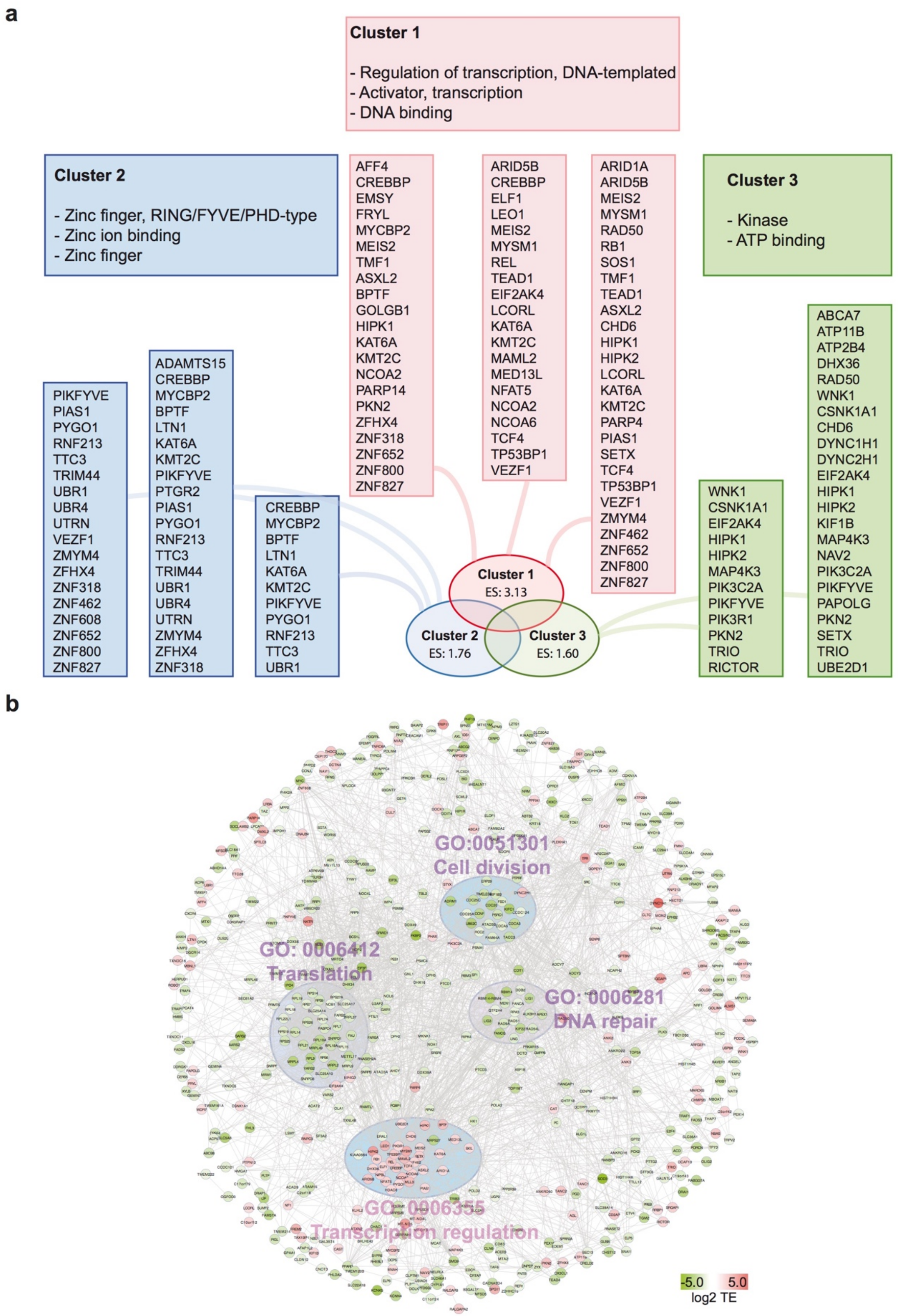

Supplementary Figure 5. Gene ontology and network analysis of translationally regulated mRNAs identified from polysome profiling. (a) DAVID biological function cluster analysis.

Top three clusters with highest enrichment scores (ES) were shown according to their GO functional annotations with the list of genes enriched in each cluster. (b) STRING protein functional interaction network of the candidate mRNAs that are regulated at the translational level in drug-tolerant persister cells. TE: translation efficiency.

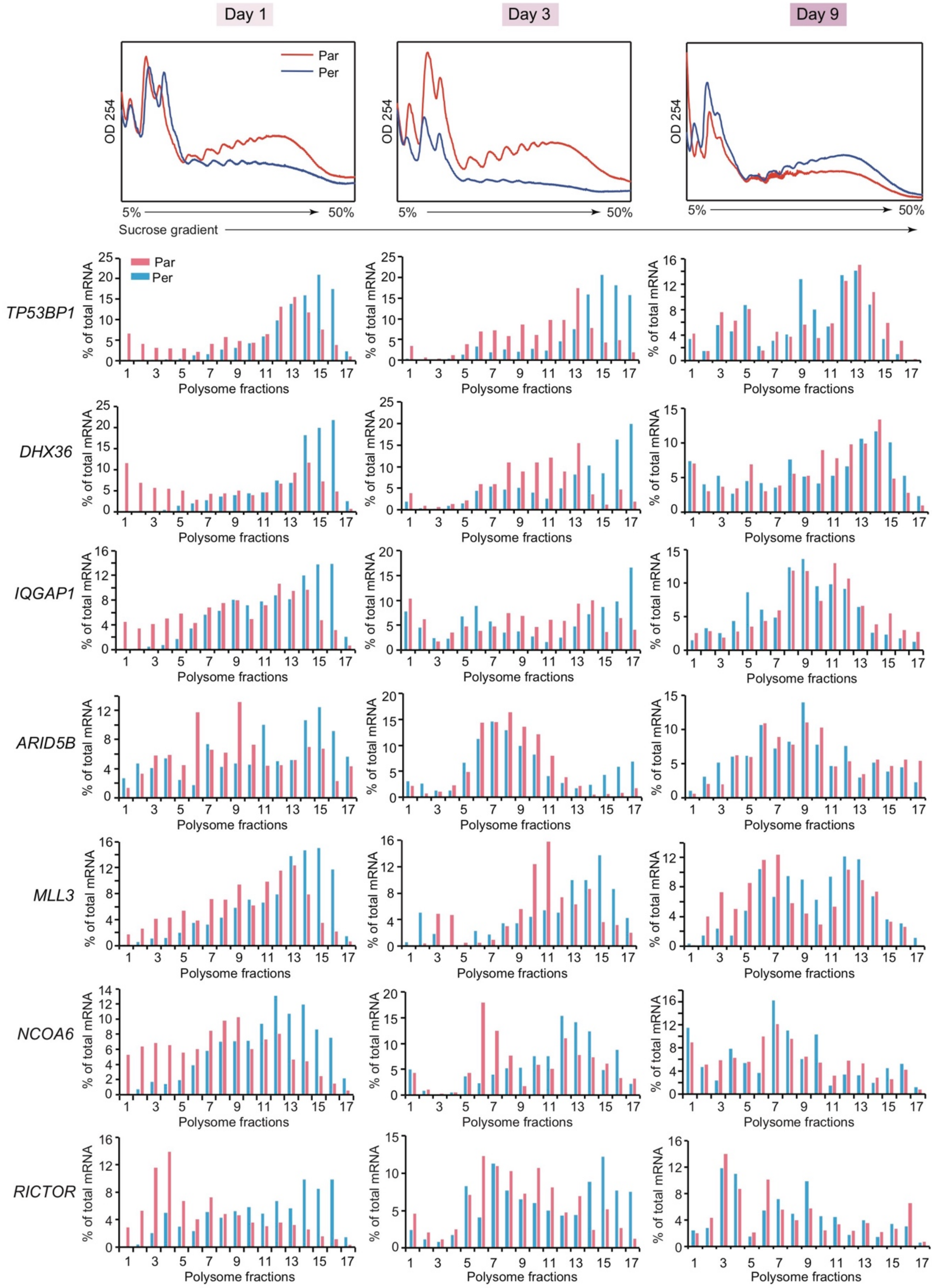

Supplementary Figure 6. RT-qPCR validation of translation activity of candidate mRNAs extracted from each polysome fraction. A375 parental and persister cells were subjected to polysome profiling on day 1, 3 and 9 after recovery from initial BRAFi/MEKi combination

treatment. The quantity of RNAs in each fraction was evaluated by RT-qPCR. RNAs from each fraction (1 - 17) correspond to free RNAs (fraction 1-5) and ribosome-bound RNAs (fraction 6-17). Highly translated RNAs are bound to more ribosomes (polysomes, fraction 13-17). RNAs found in polysome fractions are the ones with higher translation efficiency. This is the case for all the mRNAs tested on day 1 in persister cells.

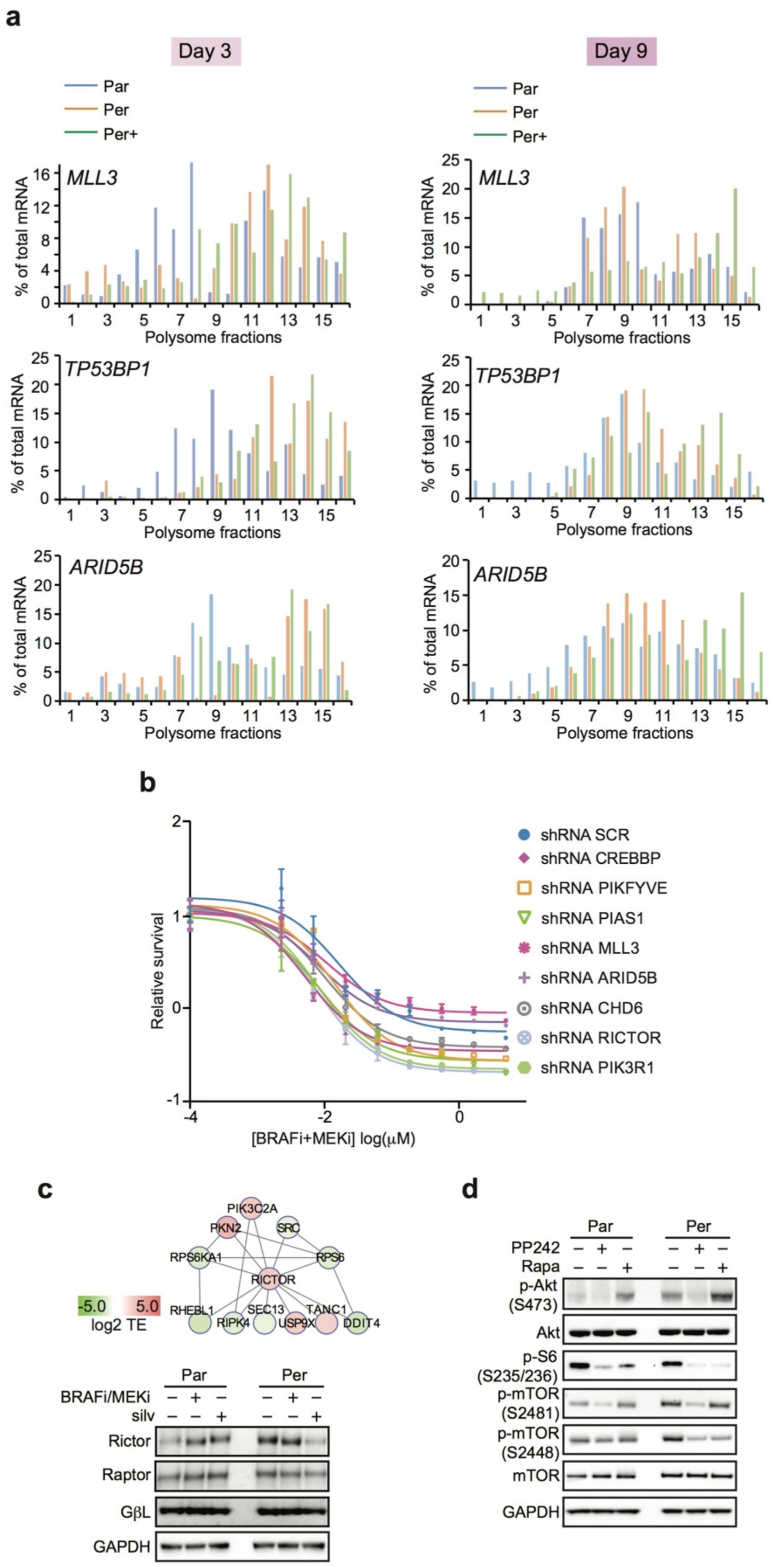

Supplementary Figure 7. Translation remodelling is correlated with persistent state in which histone modification epigenetic enzyme and mTORC2-related pathways are involved in persister cell survival. (a) A375 parental cells (Par) were treated with BRAFi and MEKi during 72 h. Drug-tolerant persister cells were recovered and divided into two groups: (i) Persister

(Per): persister cells were re-cultured in drug-free medium during 9 days; (ii) Persister (Per+): persistent cells were continually exposed to PLX4032 (1 μM) and Cobimetinib (1 μM). Polysome profiling was performed on day 3 and day 9, and RNAs were extracted from each fraction. RT-qPCR was performed to quantify the distribution of each candidate mRNA in each polysome fraction. A375 parental cells were used as a control. (b) A375 cells expressing each individual shRNA were treated with PLX4032 and Cobimetinib with indicated concentrations, and cell viability was analysed by the WST-1 viability assay. (c) Upper panel: STRING network analysis of RICTOR interaction proteins in polysome profiling. Nodes were colored according to their logarithm 2 of translation efficiency (log2TE). Lower panel: Western blotting analysis of the mTORC1 and mTORC2 principal components. A375 parental and persister cells were treated with BRAFi/MEKi or eIF4Ai at indicated concentrations for 6 hours. GAPDH was used as a loading control. Silv: silvestrol; PLX: PLX4032; Cobi: Cobimetinib. (d) Western blotting analysis of mTOR/AKT signaling pathway components. A375 parental and persister cells were treated with mTORC1 and mTORC2 ATPase inhibitor (PP242) or mTORC1 allosteric inhibitor (Rapamycin, rapa) at indicated concentrations for 4 hours. GAPDH was used as a loading control. The raw data of Supplementary Figure 7c and 7d are available in Source Data.

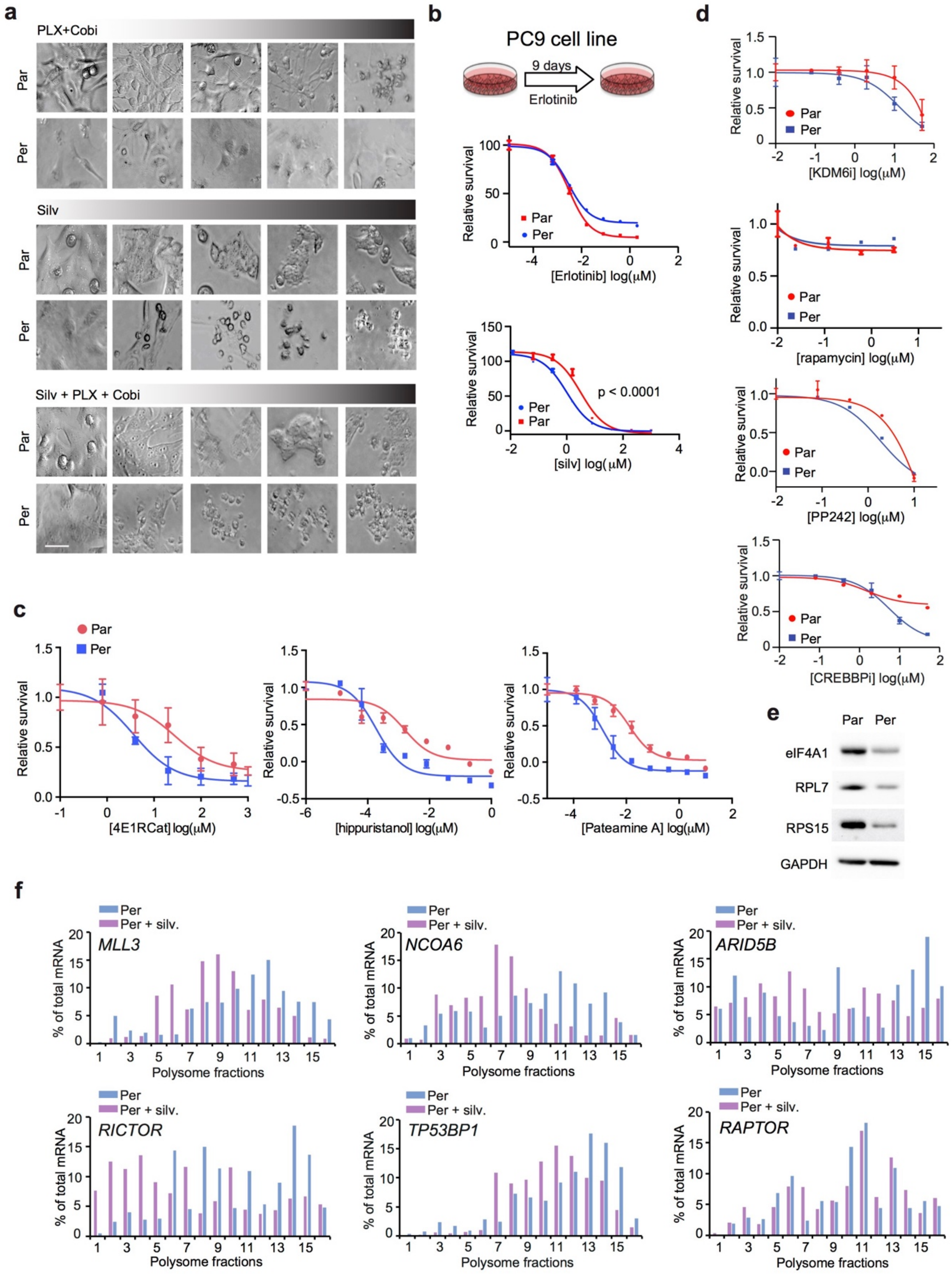

Supplementary Figure 8. eIF4Ai inhibits the translation remodelling and selectively sensitizes persister cells. (a) Drug-tolerant persister cells were generated by 72-h treatment of BRAFi/MEKi. On the second day after the recovery of persistent cells, both parental and

persistent cells were treated with the indicated treatment for 48 h, and cell viability was evaluated using WST-1. Representative cell images are shown. Top panel: PLX4032 + Cobimetinib; middle panel: silvestrol; bottom panel: PLX4032 + Cobimetinib + silvestrol. Scale bar: 10μm. (b) PC9 non-small cell lung cancer cells were treated with erlotinib (2.5 μM) for 9 days to generate drug tolerant persister cells (Per). Both PC9 parental and DTP cells were then re-challenged with erlotinib or silvestrol at indicated concentrations. ANOVA analysis was used to calculate the p-value. (c) WST1-based cell viability assay in persister versus parental cells treated with different translation initiation inhibitors. (d) WST1-based cell viability assay in persister versus parental cells treated with the indicated inhibitors. (e) Western blot analysis of eIF4A and ribosomal proteins in parental and persister cells. (f) Up-regulation of the translation efficiency of the candidate mRNAs was abrogated by eIF4A inhibition. A375 drug-tolerant persister cells were treated with silvestrol (silv, 30 nM) for 4 h and subjected to polysome profiling. HPRT was used as a control because its translation is independent of eIF4A. The raw data of Supplementary Figure 8b, 8c, 8d and 8e are available in Source Data.

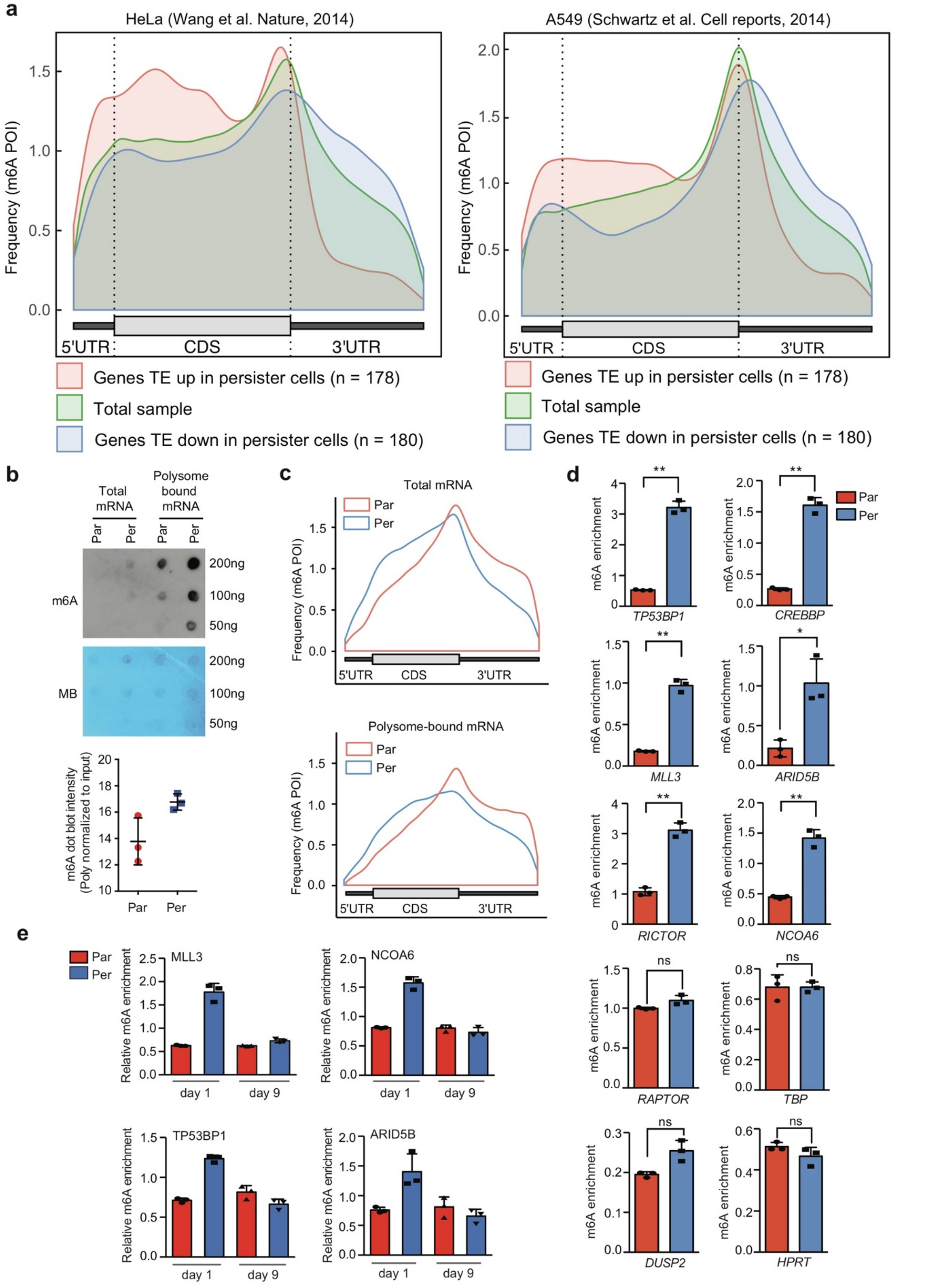

Supplementary Figure 9. Translationally up-regulated mRNAs showed higher m^6A enrichment in polysome fractions of persister cells. (a) The distribution of m6A peaks for mRNAs up-

regulated (red) and down-regulated (blue) at the translational level in persister cells. The whole population of mRNAs was plotted in green as a control. (b) Top panel: Dot blot analysis of m^6A in mRNAs extracted from total lysates and polysome fractions in persister versus parental cells. Methylene blue (MB) staining was used as loading control. Bottom panel: semi-quantification of the m^6A dot blot. (c) Metagene profiles of enrichment of m^6A modifications across mRNA transcriptome of parental and persister cells. (d) qRT-PCR analysis of m6A antibody-based RNA immunoprecipitation in persister versus parental cells with specific primers against indicated genes (n = 3, **, p-value <0.01, unpaired t-test). (e) qRT-PCR quantification of m6A enrichment in polysome-bound mRNA at indicated time points.

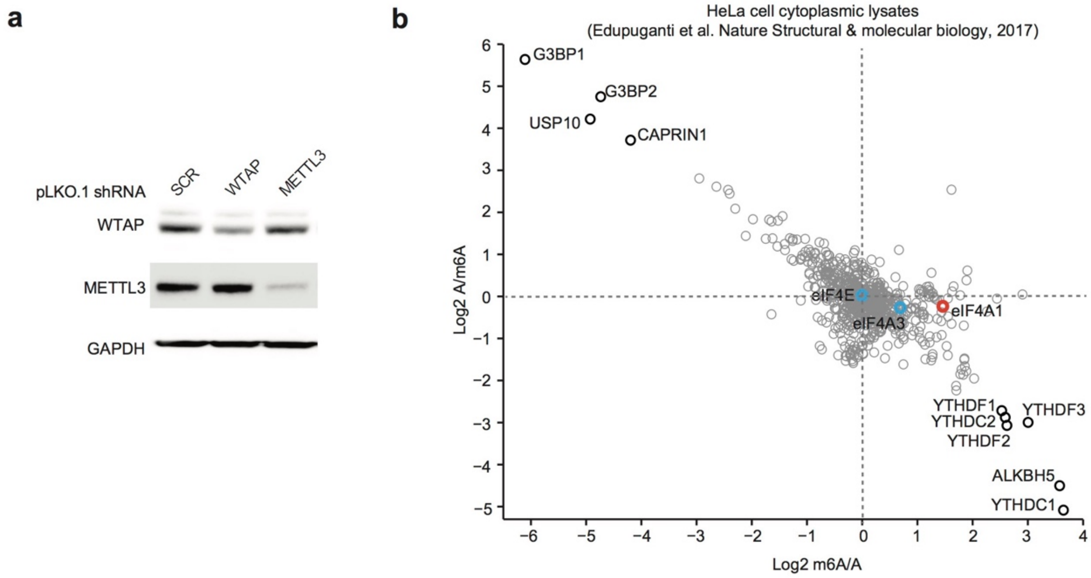

Supplementary Figure 10. m^6A enrichment is reversible and eIF4Ai decreases the m^6A enrichment in polysome-bound mRNAs in persister cells. (a) Western blot analysis of the efficiency of the shRNA knockdowns of m6A methyltransferase in A375 melanoma cells. (b) Data were obtained from Edupuganti et al. 2017[42] and plotted with the log2 ratio of m^6A/A against log2 ratio of A/m^6A. Lower right quadruplet showed protein enrichment on m^6A sequences. The raw data of Supplementary Figure 10a are available in Source Data.
