# Supplementary material for: An epitranscriptomic mechanism underlies selective mRNA translation remodelling in melanoma persister cells
Source: Nat Commun. 2019 Dec 16;10:5713. doi: 10.1038/s41467-019-13360-6 (PMC6915789; doi:10.1038/s41467-019-13360-6)
Supplement: Supplementary file 3 — Reporting Summary [file 41467_2019_13360_MOESM3_ESM.pdf]

nature research

Corresponding author(s): DBPR NCOMMS-18-25639A

Last updated by author(s): Sep 25, 2019

Reporting Summary

Nature Research wishes to improve the reproducibility of the work that we publish. This form provides structure for consistency and transparency in reporting. For further information on Nature Research policies, see Authors & Referees and the Editorial Policy Checklist .

Statistics

For all statistical analyses, confirm that the following items are present in the figure legend, table legend, main text, or Methods section.

n/a	Confirmed	
☐	☒	The exact sample size (*n*) for each experimental group/condition, given as a discrete number and unit of measurement
☐	☒	A statement on whether measurements were taken from distinct samples or whether the same sample was measured repeatedly
☐	☒	The statistical test(s) used AND whether they are one- or two-sided<br>*Only common tests should be described solely by name; describe more complex techniques in the Methods section.*
☒	☐	A description of all covariates tested
☒	☐	A description of any assumptions or corrections, such as tests of normality and adjustment for multiple comparisons
☐	☒	A full description of the statistical parameters including central tendency (e.g. means) or other basic estimates (e.g. regression coefficient) AND variation (e.g. standard deviation) or associated estimates of uncertainty (e.g. confidence intervals)
☐	☒	For null hypothesis testing, the test statistic (e.g. *F*, *t*, *r*) with confidence intervals, effect sizes, degrees of freedom and *P* value noted<br>*Give P values as exact values whenever suitable.*
☒	☐	For Bayesian analysis, information on the choice of priors and Markov chain Monte Carlo settings
☒	☐	For hierarchical and complex designs, identification of the appropriate level for tests and full reporting of outcomes
☒	☐	Estimates of effect sizes (e.g. Cohen's *d*, Pearson's *r*), indicating how they were calculated

Our web collection on statistics for biologists contains articles on many of the points above.

Software and code

Policy information about availability of computer code

Data collection	Affymetrix expression console software
Data analysis	Whole-exome sequencing data was analysed by Rstudio and MuTect. Clariom D human exon-array data was analysed by Xtail and Cytoscape. m6A-seq and relative m6A-seq public dataset were analysed by ExomePeak and Guitar R. Polysome profile data was analysed by Matlab. m6A dot blot data was analysed by Image J.

For manuscripts utilizing custom algorithms or software that are central to the research but not yet described in published literature, software must be made available to editors/reviewers. We strongly encourage code deposition in a community repository (e.g. GitHub). See the Nature Research guidelines for submitting code & software for further information.

Data

Policy information about availability of data

All manuscripts must include a data availability statement. This statement should provide the following information, where applicable:

- Accession codes, unique identifiers, or web links for publicly available datasets
- A list of figures that have associated raw data
- A description of any restrictions on data availability

Raw Sequencing data will be available in the GEO database for Affymetrix exon array data and m6A-seq data under the accesion number GSE137726. Whole exome sequencing data are available in Sequence Read Archive under the accession number PRJNA573468. The source data underlying Figure 1b, Figure 2d, e, f, g, Figure 3b, c, e, f, g, i, Figure 4e, g, Figure 5a, c, d and Sup Fig. 1c, d, Sup Fig. 2b, Sup Fig. 3a, Sup Fig. 7a, c, d, Sup Fig. 8b, c, d, e, f and Sup Fig. 10a are provided as a Source Data file. All relevant data can also be inquired from the authors.

Field-specific reporting

Please select the one below that is the best fit for your research. If you are not sure, read the appropriate sections before making your selection.

☒ Life sciences ☐ Behavioural & social sciences ☐ Ecological, evolutionary & environmental sciences

For a reference copy of the document with all sections, see nature.com/documents/nr-reporting-summary-flat.pdf

Life sciences study design

All studies must disclose on these points even when the disclosure is negative.

Sample size	No sample size calculation was performed beforehand. Sample sizes were chosen according to established practice in the field.
Data exclusions	No data point has been excluded.
Replication	For reproducibility biological replicates were performed on different days and different cell lines.
Randomization	n.a.
Blinding	n.a.

Reporting for specific materials, systems and methods

We require information from authors about some types of materials, experimental systems and methods used in many studies. Here, indicate whether each material, system or method listed is relevant to your study. If you are not sure if a list item applies to your research, read the appropriate section before selecting a response.

Materials & experimental systems

n/a	Involved in the study	
☐	☒	Antibodies
☐	☒	Eukaryotic cell lines
☒	☐	Palaeontology
☒	☐	Animals and other organisms
☒	☐	Human research participants
☒	☐	Clinical data

Methods

n/a	Involved in the study	
☒	☐	ChIP-seq
☐	☒	Flow cytometry
☒	☐	MRI-based neuroimaging

Antibodies

Antibodies used	see the section of Methods
Validation	only commercial and validated antibodies have been used.

Eukaryotic cell lines

Policy information about cell lines

Cell line source(s)	All cell lines are purchased from ATCC except the ones as indicated in the section of Methods.
Authentication	n.a.
Mycoplasma contamination	Cell lines, except non-small cell lung cancer cell line PC9, were tested for mycoplasma contamination by PCR-based method.
Commonly misidentified lines (See ICLAC register)	no commonly misidentified lines have been used

Flow Cytometry

Plots

Confirm that:

- [x] The axis labels state the marker and fluorochrome used (e.g. CD4-FITC).
- [x] The axis scales are clearly visible. Include numbers along axes only for bottom left plot of group (a 'group' is an analysis of identical markers).
- [x] All plots are contour plots with outliers or pseudocolor plots.
- [x] A numerical value for number of cells or percentage (with statistics) is provided.

Methodology

Sample preparation	A375 cells were treated with BRAFi/MEKi for 3 days. The rest of survival cells were collected, termed persister cells. The parental cells (never exposed to BRAFi/MEKi) and persister cells were stained with Caspase3/7 kit (Thermo, #C10423).
Instrument	FACS LSR II (Becton Dickinson)
Software	Matlab
Cell population abundance	Cells that were not stained with caspase3/7 kit were used as control to gate and plot the histogram of the positive population.
Gating strategy	FSC-A vs SSC-A (debris exclusion)

- [ ] Tick this box to confirm that a figure exemplifying the gating strategy is provided in the Supplementary Information.
