# Supplementary material for: An epitranscriptomic mechanism underlies selective mRNA translation remodelling in melanoma persister cells
Source: Nat Commun. 2019 Dec 16;10:5713. doi: 10.1038/s41467-019-13360-6 (PMC6915789; doi:10.1038/s41467-019-13360-6)
Supplement: Supplementary file 4 — Description of Additional Supplementary Files [file 41467_2019_13360_MOESM4_ESM.docx]

**Description of Additional Supplementary Files**

File name: Supplementary Data 1

Description: Dataset of the translationally upregulated mRNAs identified by polysome profiling in persister cells.

File name: Supplementary Data 2

Description: Dataset of the translationally downregulated mRNAs identified by polysome profiling in persister cells.

File name: Supplementary Data 3

Description: Chemical structures, targets and response curves of the panel of small-molecule compounds used in this study.

File name: Supplementary Data 4

Description: m^6^A sequencing public dataset used in the study.
