# Supplementary material for: An epitranscriptomic mechanism underlies selective mRNA translation remodelling in melanoma persister cells
Source: Nat Commun. 2019 Dec 16;10:5713. doi: 10.1038/s41467-019-13360-6 (PMC6915789; doi:10.1038/s41467-019-13360-6)
Supplement: Supplementary file 7 — Supplementary Data 3 [file 41467_2019_13360_MOESM7_ESM.pdf]

Extended Data Table 3 - The chemical structures, the targets and the response curves of the panel of small-molecule compounds. A375 parental cells and persistent cells were treated with the compounds at the indicated concentrations for 48 h. The viability of cells was assayed using WST-1.

Compound Name	Target	Structure	WST-1 dose curve
VX680	Aurora kinase	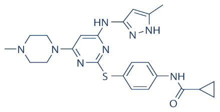	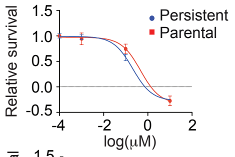
Vemurafenib	BRAFV600E	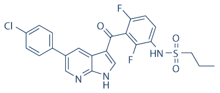	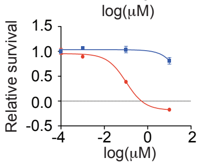
Sunitinib	KIT<br>FLT3<br>PDGFRβ<br>FGFR1	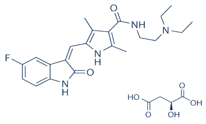	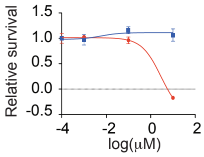
Silvestrol	eIF4A	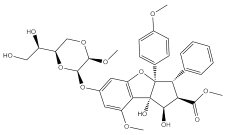	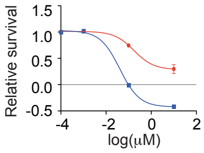
SB743921	Kinesin spindle protein	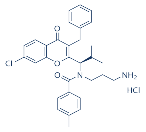	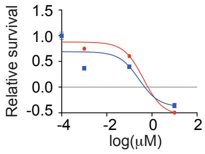
PP242	mTOR	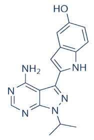	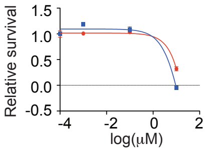
PU-H71	HSP90	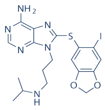	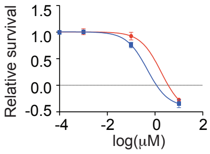
PF670462	CK1ε	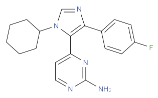	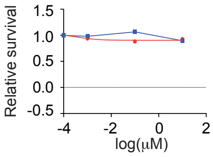
Olaparib	PARP1/2	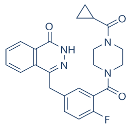	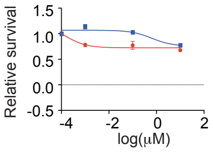

<table>
<tr><th>Compound Name</th><th>Target</th><th>Structure</th><th>WST-1 dose curve</th></tr>
<tr><td>Nifuroxazide</td><td>STAT3</td><td>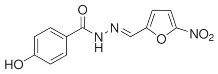</td><td>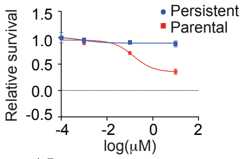
</td></tr>
<tr><td>MG132</td><td>Proteasome</td><td>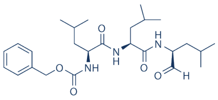</td><td>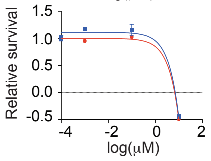
</td></tr>
<tr><td>LY294002</td><td>PI3Kα/δ/β</td><td>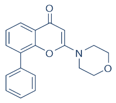</td><td>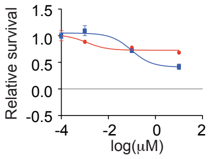
</td></tr>
<tr><td>Imatinib</td><td>v-Abl
c-KIT
PDGFR</td><td>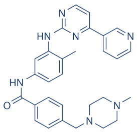</td><td>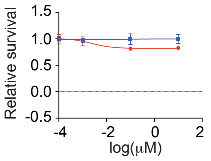
</td></tr>
<tr><td>Fludarabine</td><td>STAT1</td><td>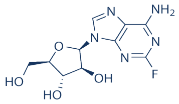</td><td>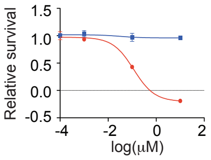
</td></tr>
<tr><td>Crizotinib</td><td>c-MET</td><td>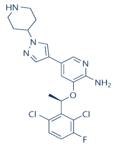</td><td>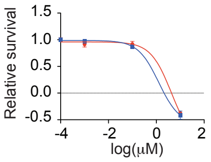
</td></tr>
<tr><td>Cobimetinib</td><td>MEK1</td><td>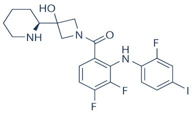</td><td>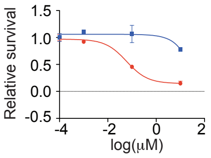
</td></tr>
<tr><td>Cilomilast</td><td>PDE4</td><td>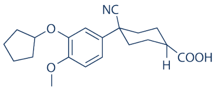</td><td>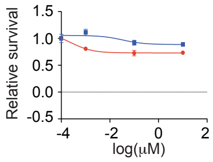
</td></tr>
<tr><td>Capmatinib</td><td>c-MET</td><td>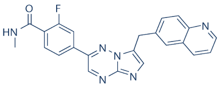</td><td>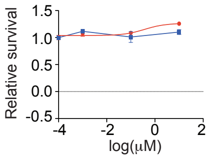
</td></tr>
</table>

Compound Name	Target	Structure	WST-1 dose curve
Bisindoylmaleimide II	PKC	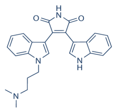	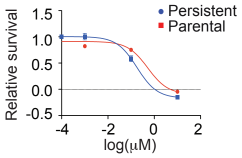
Bafilomycin A1	H+ ATPase	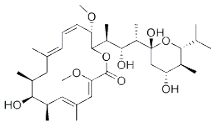	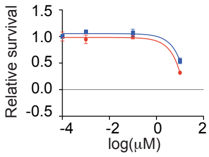
AZD2858	GSK-3	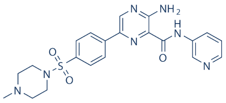	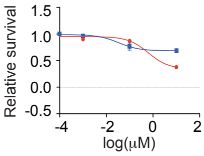
AT7519	CDK1/2/4/6/9	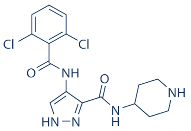	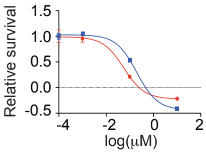
Aciclovir	DNA polymerase	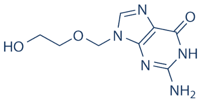	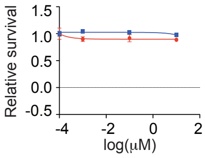
Myriocin	sphingosine biosynthesis	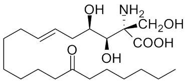	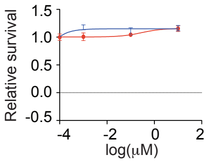
