# Supplementary material for: An epitranscriptomic mechanism underlies selective mRNA translation remodelling in melanoma persister cells
Source: Nat Commun. 2019 Dec 16;10:5713. doi: 10.1038/s41467-019-13360-6 (PMC6915789; doi:10.1038/s41467-019-13360-6)
Supplement: Supplementary file 2 — Peer Review [file 41467_2019_13360_MOESM2_ESM.pdf]

Reviewers' comments:

Reviewer #1 (Remarks to the Author):

The authors present a large body of work detailing new findings that implicate eIF4A1-dependent, selective mRNA re-modelling in the emergence of melanoma persister cells following B-raf/MEK inhibitor treatment. The data generally justify the conclusions drawn and convincingly make that case for eIF4A-dependent alterations in the translation of a subset of mRNAs as a critical parameter of persistence following B-raf/MEKi based therapy. The data further suggest that the combination of 4A inhibition with B-raf/MEK inhibition might be a potent means to defeating the emergence of persisters.

Specific Comments:

1) line 77-78- it may be a bit of an overreach to suggest that these persister cells serve as a reservoir for the development of genetically-resistant cells. Could be true but this would require more definitive experimentation to tackle this specific point- though this point is not directly germane to the central thesis of the manuscript.
2) Lines 125-6 contend a specific effect on a subset of mRNAs though the data are clearest for CREBBP specifically. The subset shown in supple figure 6 seem to display a variety of patterns not entirely consistent with the pattern clearly evident for CREBBP.
3) Data for MLL3 are inconsistent between supplemental figures 6 and 7.
4) There are no polysome profile data for RICTOR shown but it seems RICTOR is considered part of the subset of mRNAs?
5) Figure Legend 2- in the figure legend, define Par, Per and Per+
6) Line 133- add a "." between "treatment" and "we". Start a new sentence with "We"
7) Line 130- change "deviated" to "deviating"
8) line 161- effects on H3K9Ac, H3K4me1 and H3K27me3 are so marginal that it seems these could be excluded from the manuscript.
9) line 165- mTORser2448 is also elevated but for some reason not mentioned.
10) Paragraph starting on line 174- the whole paragraph could be re-written as all of the drugs used show only marginal selective killing of persisters. The point of the paragraph is that 4A inhibition does show selective killing but the structure of the paragraph clouds this point.
11) Suppl Fig 8C is unnecessary- consider excluding
12) Line 191- shRNA knockdown of eIF4A1 but not eIF4A2 or 3 selectively inhibits the emergence of persisters. Please comment on the specificity for 4A1 vs 4A2 and 4A3. Consider elevating supplemental fig 8d to a bona fide figure, not supplemental.
13) Line 280- change "seemly" to "seemingly"
14) Line 283-284- the statement that 4A1i showed a much stronger effect on persisters than parental cells is untrue based upon the data shown in Figure 4i. 4A1i profoundly decreased the m6A level in polysomal fractions in both parental and persister cells.
15) Line 299- "to directly recruits" should be "to recruit directly"
16) Figure 4e- please consider normalizing the m6A polysome bound mRNA to total mRNA as the total is elevated.
17) Consider adding supplemental fig 10c to the regular figure 4J or as 4K.

Reviewer #2 (Remarks to the Author):

This study by Shen et al. suggests a novel non-genomic mechanism supporting the persistence of cancer cells to targeted therapy. According to the model suggested by the authors, m6A modifications and eIF4A regulate translational remodeling of BRAFV600 mutant melanoma cells surviving BRAF/MEK inhibition. In light of this finding, the authors suggest that targeting of translation initiation may serve as a novel therapeutic approach to prevent acquired drug resistance.

They provide compelling evidence for drug-dependent translational remodeling of persister cells by

showing reversible changes in their translation activity compared to parental non-treated cells. By comparing the polysome profiles of drug-tolerant persister cells to parental cells, it is evident that global translation is reduced in persister cells, and this attenuation is reversible after a period of drug-free culture. Further genome-wide analysis of cytoplasmic and polysomal mRNA by exon-array identified a subset of translationally up-regulated genes in persister cells. The authors convincingly show the association of this translational up-regulation with a reversible persister cell state by demonstrating that translation efficiency reverts back to baseline upon drug withdrawal. Analysis of the biological function of these genes emphasized their involvement in multiple regulatory pathways including epigenetic remodeling and PI3K signaling.

The authors address the relation of this gene-specific increase in translation to drug resistance by shRNA-mediated knockdown of 30 selected genes and downstream analysis of persister cell survival. This screen revealed genes whose knockdown led to reduced persister cell survival in response to lethal concentrations of BRAF/MEK inhibitors. This evidently links some of the translationally up-regulated mRNAs to persister cell survival.

To test whether small-molecule compounds can target persister cells, the researchers performed a small-scale screen and revealed an increased sensitivity of persister cells to eIF4A inhibition. This fits with the global reduction in translation -with specific translational upregulation of key transcripts - observed in persister cells compared to parental cells. It is therefore likely that persister cells are more sensitive to further attenuation of translation activity essential for their survival. It would be interesting to explore whether other components of the translation initiation machinery are essential for persister cell survival.

The authors further explore which mRNA features may drive selective translation in persister cells. The data provided herein points to an m6A-related mechanism. By performing an m6A dot-blot assay on polysome-bound mRNA, the authors found an enrichment of m6A modifications in persister cells compared to parental cells. They further demonstrate that this enrichment is found in mRNAs that are more efficiently translated in persister cells. Importantly, this enrichment is absent when examining translationally down-regulated and housekeeping genes. As these results suggest an m6A-assosiated selective regulation of translation efficiency, it would be interesting to perform a perturbation of the m6A writer METTL3 in parental and persister cells, and examine whether the up-regulation in translation efficiency of specific genes is affected. It remains to be established how m6A is distributed along the translationally up-regulated transcripts in persister cells compared to parental cells, and by which mechanism it may direct the specific translation of mRNAs in response to targeted therapy.

All in all, this is a very interesting and novel study that was rigorously performed. Results are carefully interpreted and not overstated. In my opinion, the m6A mechanistic link is quite well established and importantly is also in line with other recent studies in the field linking 5'UTR m6A with translation. However. I would like to see at least one additional piece of orthogonal evidence – for example, manipulation of m6A levels by Mettl3/14 knockdown. In addition, the authors should perform a simple m6A-seq experiment to prove that m6A is indeed enriched in the 5’ UTR of translationally-upregulated transcripts. Other than that, I fully support publication of this fine study.

Reviewer #3 (Remarks to the Author):

This manuscript ‘An epitranscriptomic mechanism underlies selective mRNA translation remodelling in melanoma persister cells’ by Shen et al., describes the changes in mRNA translation that accompany

the short-term and reversible resistance of (BRAFV600 mutant) human cancer cells to pharmacological inhibition of the MAPK pathway (BRAFi/MEKi) and explores the underlying mechanism. The authors begin by describing and characterizing a subpopulation of 'persister' (Per) cells that can withstand MEKi. Next they find a global reduction in nascent polypeptide synthesis in Per cells that corresponds with a dramatic decrease in global translation as measured by polysome profiling using sucrose gradients. Next, they used microarrays to globally analyze changes in mRNA translation efficiency (TE) and identify that while many genes have lower TE in the Per cells compared to the parental (Par) control cells, there is a small subset of mRNAs that have increased TE in Per cells. This group of mRNAs is enriched for certain chromatin modifiers and stress-responsive kinases. shRNA-mediated depletion of 30 of these genes revealed that each of these individual genes has a modest impact on the frequency of PER cells. Testing a small panel of small-molecule inhibitors they found that Silvestrol, a known eIF4A inhibitor, selectively suppressed the growth of Per cells compared to Par cells. Moreover, expression of the mRNAs with TE upregulated in Per cells was found to be sensitive to Silvestrol. Interestingly the authors showed that eIF4Ai in combination with BRAFi/MEKi more effectively inhibited cancer cell viability (colony formation) that either inhibitor alone. Finally, the authors interrogate the features of the mRNAs with increased TE in Per cells in an attempt to understand how translation of this subset of mRNAs might be distinguished and regulated differently from bulk mRNAs in Per cells. They find no difference in 5' UTR length, or minimum free energy between TE up- or down- regulated genes, and a very small difference in GC-content. However their re-analysis of published m6A meRIP-Seq data suggests that the mRNAs with increased TE are enriched for m6A marks in their 5'UTRs and the 5'-end of the CDS and have less m6A in their 3' UTRs compared to either TE-down or total mRNAs suggesting that m6A might somehow be involved in the regulation of the TE-up mRNAs in Per cells.

Overall, this is a very interesting study. While much of the work is performed to an excellent standard and the results convincing, there remain some important questions that need to be addressed prior to publication in Nature Communications. In particular the possible connection between m6a, eIF4A1, and increased TE is quite weak and needs further verification.

Major concerns:'
1) Figure 4C (and S9a-b). Are the apparent differences in the m6A distribution between the sets of mRNAs related to differences in the relative mRNA abundance? In other words is the mRNA abundance of the TE-up genes more or less than the rest of the mRNAs (or the TE downregulated mRNAs) and could this represent a technical artifact that might account for these differences in the distribution of m6A across the mRNA? Also, it is unclear why the authors picked only the top 10% downregulated mRNAs and not the entire group of TE-down for this analysis. This could be addressed with more bioinformatics analysis showing the read number (mean and distribution) for each of the gene sets.

2) Figure 4d and S9b. Considering the very dramatic decrease in the polysome peak it is surprising that only 1,287 genes were translationally downregulated more than 2-fold. I would have expected many more mRNAs with decreased TE in Per cells. Also, with this large decrease in polysome peak it is expected that a corresponding increase in 80S peak or at least in subpolysome fraction should be observed. Related to this, in Figure 4i there is big increase in the 80s peak in the Silvestrol-treated cells for both Par and Per cells. However the Polysome peak in Per (- Silv) is similar to Par (+ Silv), but the 80S peak in Per (- Silv) did not increase as much as Par (+ Silv). The most likely explanation for this result is that fewer Per cells (or a lower concentration of cell lysate) was used for the polysome profiling of Per cells compared to the Par cells.

3) In general dot blots are considered of limited use for relative m6A quantification (Figures 4e-j). Ideally, this would be done by HPLC/Mass Spec which is a more quantitative and reliable way of measuring m6A levels. Moreover, in these examples the MB-staining is barely visible and so it not possible to know whether there is equal loading of RNA on these blots. More importantly, the authors incorrectly conclude that the increased level of m6A in the polysome bound mRNAs in Per cells compared to the Par cells (Figure 4e). The statement in the results section that "A strong enrichment

of m6A modification in mRNAs from heavy polysome fractions was found in persister cells compared to parental cells, with only a marginal increase observed in total mRNAs (Fig 4e)” does not appear to be the case since from the figure it seems that the relative ratio of m6A between Par and Per in the total RNA samples is quite similar to the m6A ratio in Polysome bound mRNAs from Par compared to Per cells. Therefore, the dot plot appears to show that the m6A level is increased in Per cells and that actively translating mRNAs harbor m6A marks but that the apparent increased m6A in polysomes simply reflects the increased m6A in the input RNAs from Per cells. It even seems that the increased m6A levels (in Per compared to Par) is very variable between different experiments (i.e. shown in figures 4e, figure 4i, and S9b). This further underscores my concerns about the dot blot assay for this type of analysis.

4) From the literature HPRT is reported to be a non-methylated mRNA and so presumably that is why the authors used this gene as a control (though this is not explained in the manuscript). If the authors picked HRPT as a non-methylated control mRNA, then why is the m6A enrichment shown for this mRNA in Fig 4f-j?

5) The m6A data are entirely correlative and no functional results are shown. Could the authors try knocking down the m6A methyltransferase METTL3 and testing the affect on the frequency of Per cells and the expression of the TE-up (and control) genes?

Ref : NCOMMS-18-25639-T

Point-to-Point Reply

Reviewers' comments:

Reviewer #1 (Remarks to the Author):

The authors present a large body of work detailing new findings that implicate eIF4A1-dependent, selective mRNA re-modelling in the emergence of melanoma persister cells following B-raf/MEK inhibitor treatment. The data generally justify the conclusions drawn and convincingly make that case for eIF4A-dependent alterations in the translation of a subset of mRNAs as a critical parameter of persistence following B-raf/MEKi based therapy. The data further suggest that the combination of 4A inhibition with B-raf/MEK inhibition might be a potent means to defeating the emergence of persisters.

Response: We thank the reviewer’s appreciation of the convincing character of our results and of the importance and potential application of our current work. We address the specific points raised by the reviewer as follows.

Specific Comments:

1) line 77-78- it may be a bit of an overreach to suggest that these persister cells serve as a reservoir for the development of genetically-resistant cells. Could be true but this would require more definitive experimentation to tackle this specific point- though this point is not directly germane to the central thesis of the manuscript.

Response: We thank the reviewer to raise this concern on the conclusion of this experiment. We admit that, to definitively confirm that persister cells are the sources of the evolution of genetically resistant cells, several additional experiments, such as genetically barcoded persister cells, would be needed. We modified the text (Line 83) in the revised version of the manuscript to avoid over-interpretation.

However, in our current experiments, we derived short passage clones from a single parental cells to reduce pre-existing genetic heterogeneity in the parental population. Whole-exome

sequencing of the resistant cells originating from single parental-cell derived clones showed known resistance-conferring genetic mutations, such as BRAF alternative splicing, doubled kinase domain of BRAF, MAPK pathway mutations etc (as presented in Supplementary Fig. 3). This suggests that the resistant genetic variants originate from the persister cell population. Of note, similar approaches were used in two recent works concerning persister cells in non-small cell lung cancer lines (*Nature Communications*, 2016, 7, 10690; *Nature Medicine*, 2016, 22, 262–269).

2) Lines 125-6 contend a specific effect on a subset of mRNAs though the data are clearest for CREBBP specifically. The subset shown in supple figure 6 seem to display a variety of patterns not entirely consistent with the pattern clearly evident for CREBBP.

Response: We apologize for this confusion due to a mistake in the colours used in the figure. In principal, the persister cell (Per) results were represented in blue (Fig. 2d) and parental cell (Par) results were represented in red. However, the colours were mixed up in Supplementary Fig 6 (Per was marked in red, and Par was marked in blue). This has now been corrected.

3) Data for MLL3 are inconsistent between Supplementary Figs 6 and 7.

Response: We have repeated this experiment in the various polysome fractions obtained on Day 3 in both parental and persister cells. RT-qPCR of the MLL3 mRNA performed in two independent polysome profiling experiments confirmed that on Day 3, the MLL3 mRNA is more efficiently translated in persister cells. This result (also shown below) has been integrated into Supplementary Fig 6. The variations of the MLL3 mRNA distribution in the polysome fractions on Day 3 could be due to the high dynamic of the translational reprogramming in persister cells during the transition from the persister to parental state in drug-free culture.

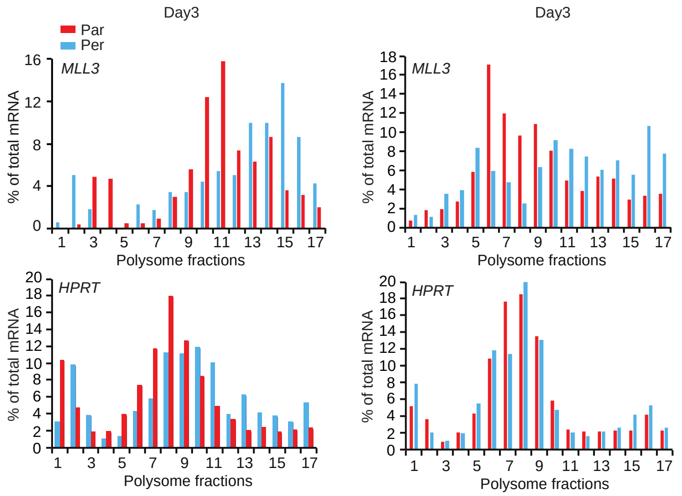

4) There are no polysome profile data for RICTOR shown but it seems RICTOR is considered part of the subset of mRNAs?

Response: We thank the reviewer for this comment, and we have now added RT-qPCR data for the RICTOR mRNA in polysome fractions taken at Day 1, 3 and 9, in both parental and persister cells. The data (also shown below) have been added in Supplementary Fig 6.

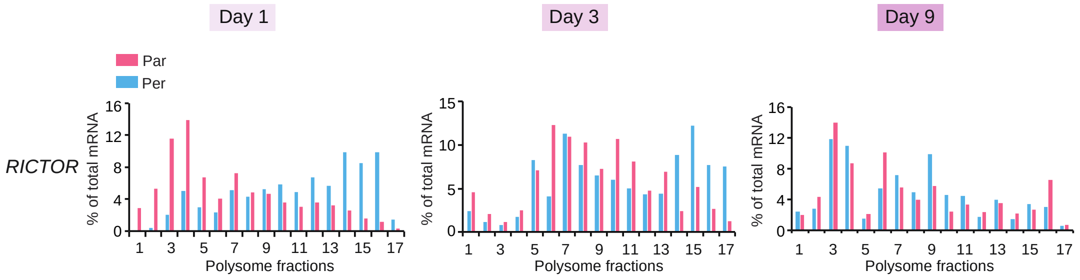

5) Figure Legend 2- in the figure legend, define Par, Per and Per+

Response: This has been added to the revised manuscript.

6) Line 133- add a "." between "treatment" and "we". Start a new sentence with "We"

Response: This has been modified according to the reviewer's suggestion.

7) Line 130- change "deviated" to "deviating"

Response: This has been corrected.

8) line 161- effects on H3K9Ac, H3K4me1 and H3K27me3 are so marginal that it seems these could be excluded from the manuscript.

Response: We have excluded these results from the revised version of the manuscript following the reviewer's suggestion.

9) line 165- mTORser2448 is also elevated but for some reason not mentioned.

Response: We have added the discussion in the revised manuscript as follows:

"**Line 164:** … We observed increased RICTOR expression (Supplementary Fig. 7c), and elevated levels of phosphorylated mTOR at serine 2481 and AKT at serine 473 in persister cells (Supplementary Fig. 7d). Serine 2481 is a marker of mTORC2 activation, which phosphorylates AKT. Phosphorylation of mTOR at serine 2448 was increased in persister cells, but to a lesser extent compared to parental cells (Supplementary Fig. 7d). This increase in persister cells may result from AKT activation since this site is PI3K/AKT-dependent. Accordingly, PP242 (a mTORC1 and mTORC2 ATPase inhibitor) abrogated both phosphor-AKT (S473) and phosphor-mTOR (S2481), whereas rapamycin (an allosteric inhibitor of mTORC1) inhibited the phosphorylation of mTOR Ser 2448 only in persister cell (Supplementary Fig. 7d).. "

10) Paragraph starting on line 174- the whole paragraph could be re-written as all of the drugs used show only marginal selective killing of persisters. The point of the paragraph is that 4A inhibition does show selective killing but the structure of the paragraph clouds this point.

Response: Following the suggestion of the reviewer, we have modified the paragraph as follows.

“**Line 179**: In an attempt to target persister cells as a therapeutic approach, we screened a panel of small-molecule compounds that target different kinases or proteins known to be involved in cancer resistance as well as various inhibitors of the pathways that were found to be up-regulated at the translational level. These data showed that silvestrol, an inhibitor of the eIF4A RNA helicase component of the eIF4F translation initiation complex, was the most selectively lethal compound towards melanoma persister cells (Fig. 3a, Supplementary Fig. 8a and Supplementary Table 3). A similar selective sensitivity to silvestrol was also observed in PC9 non-small cell lung cancer persister cells (Supplementary Fig. 8b), another well characterised persister cell model. We further evaluated the sensitivity of melanoma persister cell to three other translation initiation inhibitors, including 4E1RCat, a specific inhibitor that disrupts the eIF4E-eIF4G interaction in the eIF4F complex; hippuristanol, a compound that prevents eIF4A from binding to mRNA; and pateamine A, an inhibitor that leads to depletion of eIF4A from the eIF4F complex. All of them showed stronger toxicities on the persister cells than on the parental cells (Supplementary Fig. 8c). The sensitivity of persister cells to silvestrol was reversible upon BRAFi/MEKi withdrawal (Fig. 3b), underscoring its close correlation with the persistent state. Consistently, targeting translation remodelling directly through eIF4A inhibition was notably much more effective as compared to the effects obtained when targeting translationally up-regulated pathways, such as CREBBP (CREBBPi), H3K27m3 demethylase (KDM6i) or mTORC2 inhibitor (PP242) (Supplementary Fig. 8d)”.

11) Suppl Fig 8C is unnecessary- consider excluding

Response: Non-small cell lung cancer cell line PC9 is the most well-characterized cancer persister cell model to date (Cancer Cell. 2017, 14;32(2):221-237; Cell. 2010;141(1):69-80; Cancer Res. 2014;74(13):3579-90; Nat Chem Biol. 2016;12(7):531-8; Nature Communications, 2016, 7, 10690; Nature Medicine, 2016, 22, 262–269). We thought that showing the selective toxicity of eIF4A inhibition on this particular model would indicate that our findings can be extended beyond melanoma and would also encourage the scientist working in the field to explore the implication of translational reprogramming in other cancer types. We therefore propose to keep this data in Supplementary Fig 8b, without deviating from our main objective that focuses on melanoma persister cells.

12) Line 191- shRNA knockdown of eIF4A1 but not eIF4A2 or 3 selectively inhibits the

emergence of persisters. Please comment on the specificity for 4A1 vs 4A2 and 4A3. Consider elevating supplemental fig 8d to a bona fide figure, not supplemental.

Response: We thank the reviewer for this suggestion. We have modified Figure 3 to include the data showing the effects of knocking down the three eIF4A family members. Accordingly, we have now discussed these results in the revised manuscript as follows:

"**Line 199**: Three eIF4A proteins have been characterized in vertebrates, including eIF4A1, eIF4A2 and eIF4A3. eIF4A1 is an ATP-dependent DEAD-box RNA helicase that assists in unwinding secondary structures within the 5'-UTR of mRNAs to allow ribosome scanning. Although eIF4A1 and eIF4A2 show approximately 90 % sequence identity, they are not functionally redundant *in vivo*. For instance, eIF4A1 knockdown, which is known to induce an increase in eIF4A2, leads to a decrease of [^{35}S] methionine incorporation and global mRNA distribution in polysome profiles, showing that the up-regulation of eIF4A2 does not compensate for the reduction in mRNA translation efficiency upon eIF4A1 depletion. In addition, eIF4A1 and eIF4A2 have distinct binding partners, eIF4A1 predominantly binds to eIF4G while eIF4A2 preferentially binds to cNOT7 (a member of the CCR4-NOT complex). eIF4A3 is functionally distinct from eIF4A1 and eIF4A2 despite sharing 60 % homology. eIF4A3 is likely not involved in translation control, since it principally resides in the nucleus where it forms a key component of the exon junction complex and plays a major role in the nonsense-mediated mRNA decay. We found that knockdown of eIF4A1, but not eIF4A2 and eIF4A3, effectively inhibited the emergence of persister cells in the presence of BRAFi/MEKi (Fig 3c). ".

13) Line 280- change "seemly" to "seemingly"

Response: This has been corrected.

14) Line 283-284- the statement that 4A1i showed a much stronger effect on persisters than parental cells is untrue based upon the data shown in Figure 4i. 4A1i profoundly decreased the m6A level in polysomal fractions in both parental and persister cells.

Response: We thank the reviewer for this comment. We thereby quantified the m^{6}A intensity in the dot blot experiments. As the reviewer will appreciate, we observed a stronger

decreasing effect of eIF4A inhibition on m^{6}A enrichment in polysome fractions in persister cells than in parental cells. We have now added this result (also shown below) in Figure 6 b and c.

- m^{6}A dot blot normalization plot:

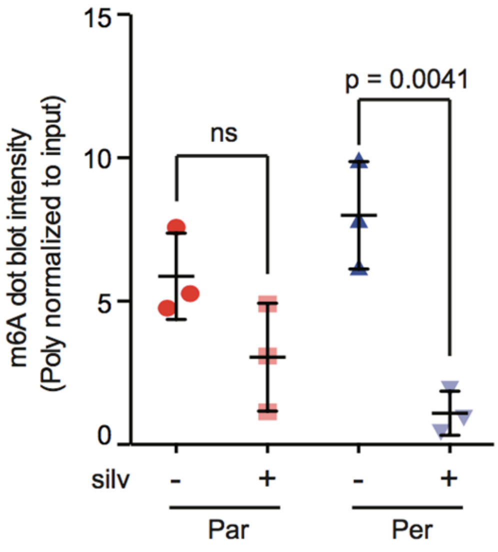

15) Line 299- "to directly recruits" should be "to recruit directly"

Response: This has been corrected.

16) Figure 4e- please consider normalizing the m6A polysome bound mRNA to total mRNA as the total is elevated.

Response: Following the suggestions of the reviewer, we quantified the intensity of the m^{6}A dot blot and methylene blue respectively. The intensity of the m^{6}A dot blot was then normalized to the methylene blue intensity, followed by normalization of polysome m6A to the total mRNA m^{6}A. In addition, we performed m^{6}A LC/MS-MS quantification experiment. The enrichment of m^{6}A was represented by normalization of m^{6}A in polysome versus m^{6}A in total RNA. These data (shown below) have now been added to the revised manuscript as Supplementary Fig. 9b and Fig. 4e.

- m^6A dot blot normalization plot:

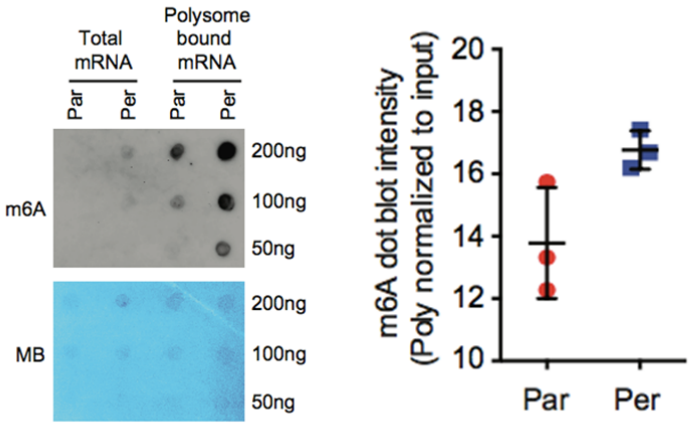

- Persister cell Day 1 m^6A LC/MS-MS quantification plot:

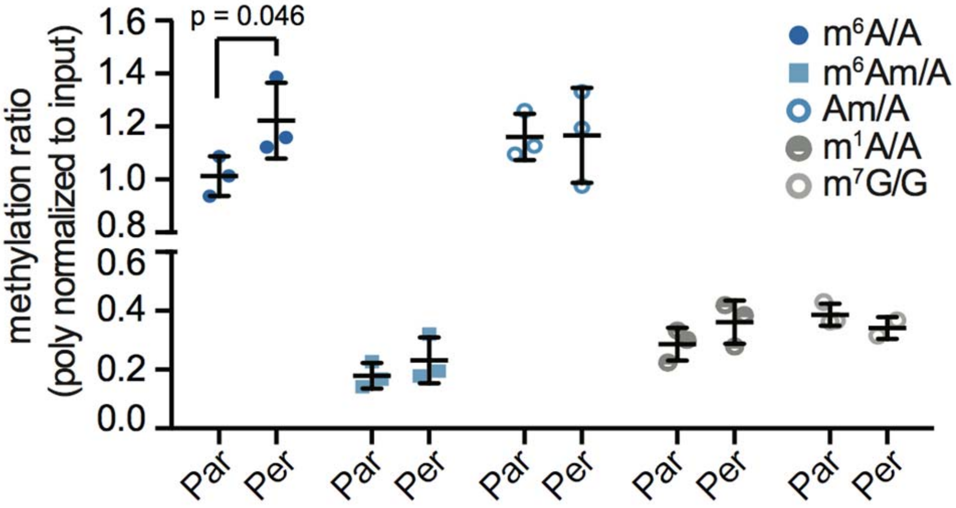

- Persister cell Day 9 (drug-free medium) m^6A LC/MS-MS quantification plot:

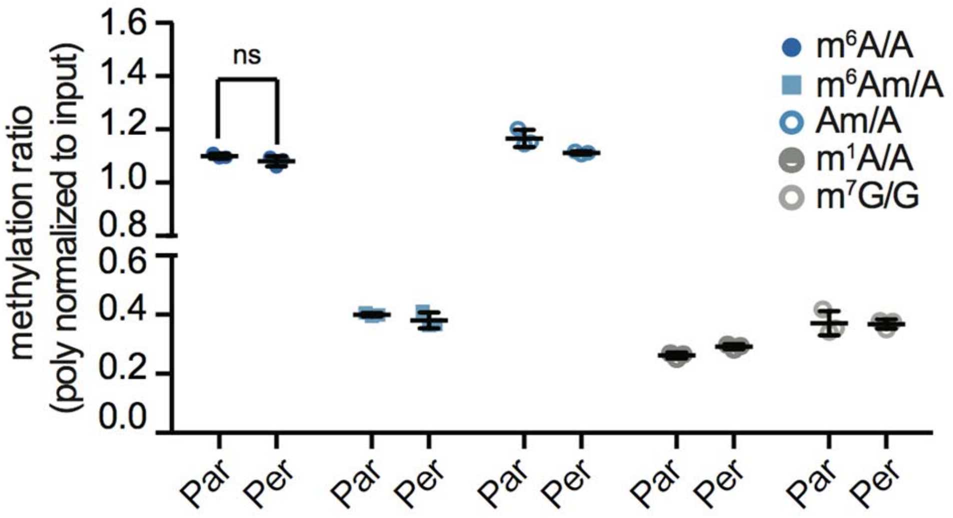

17) Consider adding supplemental fig 10c to the regular figure 4J or as 4K.

Response: We have modified the corresponding figures following the suggestions of the reviewer. These results are now added in Fig. 6d of the revised manuscript.

Reviewer #2 (Remarks to the Author):

This study by Shen et al. suggests a novel non-genomic mechanism supporting the persistence of cancer cells to targeted therapy. According to the model suggested by the authors, m6A modifications and eIF4A regulate translational remodeling of BRAFV600 mutant melanoma cells surviving BRAF/MEK inhibition. In light of this finding, the authors suggest that targeting of translation initiation may serve as a novel therapeutic approach to prevent acquired drug resistance.

They provide compelling evidence for drug-dependent translational remodeling of persister cells by showing reversible changes in their translation activity compared to parental non-treated cells. By comparing the polysome profiles of drug-tolerant persister cells to parental cells, it is evident that global translation is reduced in persister cells, and this attenuation is reversible after a period of drug-free culture. Further genome-wide analysis of cytoplasmic and polysomal mRNA by exon-array identified a subset of translationally up-regulated genes in persister cells. The authors convincingly show the association of this translational up-regulation with a reversible persister cell state by demonstrating that translation efficiency reverts back to baseline upon drug withdrawal. Analysis of the biological function of these genes emphasized their involvement in multiple regulatory pathways including epigenetic remodeling and PI3K signaling.

The authors address the relation of this gene-specific increase in translation to drug resistance by shRNA-mediated knockdown of 30 selected genes and downstream analysis of persister cell survival. This screen revealed genes whose knockdown led to reduced persister cell survival in response to lethal concentrations of BRAF/MEK inhibitors. This evidently links some of the translationally up-regulated mRNAs to persister cell survival.

To test whether small-molecule compounds can target persister cells, the researchers performed a small-scale screen and revealed an increased sensitivity of persister cells to eIF4A inhibition. This fits with the global reduction in translation -with specific translational upregulation of key transcripts - observed in persister cells compared to parental cells. It is therefore likely that persister cells are more sensitive to further attenuation of translation activity essential for their survival. It would be interesting to explore whether other components of the translation initiation machinery are essential for persister cell survival.

Response: We thank the reviewer for his/her comprehensive summary of our work and the appreciation for the importance of our results and his/her encouraging general comments.

We address his/her remarks as follows:

We evaluated the sensitivity of melanoma persister and parental cells to other small molecule inhibitors of the eIF4F complex: (i) 4E1RCat, a specific inhibitor that disrupts the eIF4E-eIF4G interaction; (ii) hippuristanol, a compound that prevents eIF4A from binding to mRNA; (iii) pateamine A, another inhibitor that leads to depletion of eIF4A from the eIF4F complex. Similar to what we observed with silvestrol, all these inhibitors showed stronger toxicities on persister cells than on parental cells. We have added these results (shown below) into the revised version of the manuscript as Supplementary Fig 8c.

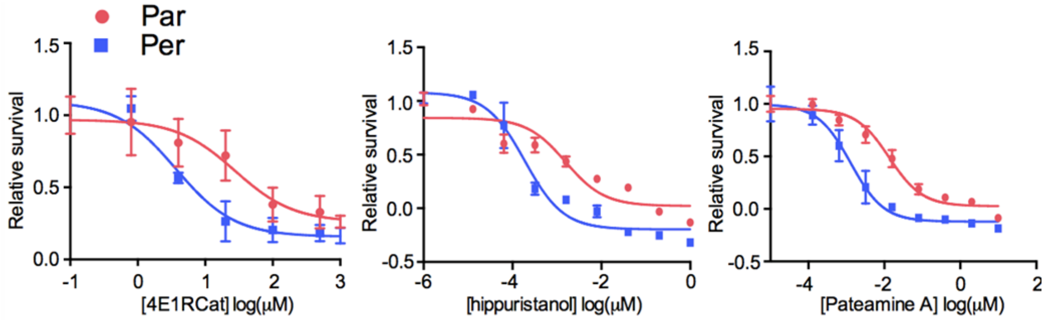

The authors further explore which mRNA features may drive selective translation in persister cells. The data provided herein points to an m6A-related mechanism. By performing an m6A dot-blot assay on polysome-bound mRNA, the authors found an enrichment of m6A modifications in persister cells compared to parental cells. They further demonstrate that this enrichment is found in mRNAs that are more efficiently translated in persister cells. Importantly, this enrichment is absent when examining translationally down-regulated and housekeeping genes. As these results suggest an m6A-assosiated selective regulation of translation efficiency, it would be interesting to perform a perturbation of the m6A writer METTL3 in parental and persister cells, and examine whether the up-regulation in translation efficiency of specific genes is affected. It remains to be established how m6A is distributed along the translationally up-regulated transcripts in persister cells compared to parental cells, and by which mechanism it may direct the specific translation of mRNAs in response to

targeted therapy. All in all, this is a very interesting and novel study that was rigorously performed. Results are carefully interpreted and not overstated. In my opinion, the m6A mechanistic link is quite well established and importantly is also in line with other recent studies in the field linking 5'UTR m6A with translation. However, I would like to see at least one additional piece of orthogonal evidence – for example, manipulation of m6A levels by Mettl3/14 knockdown.

Response: To address the m^6A regulation in an orthogonal manner, we knocked down METLL3 or WTAP by pLKO.1 shRNAs in A375 cells. We found that knockdown of METTL3 or WTAP strongly sensitizes melanoma cell exposure to BRAFi/MEKi. This result (shown below) has been added in Fig. 5a.

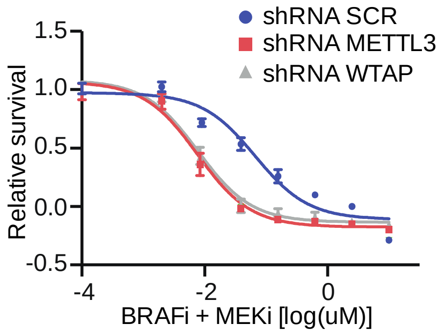

We subsequently showed that knockdown (shRNA) of either METTL3 or WTAP significantly reduced the persister cell-derived colony formation. These results (shown below) have been added in Fig. 5b, c and Supplementary Fig 10a.

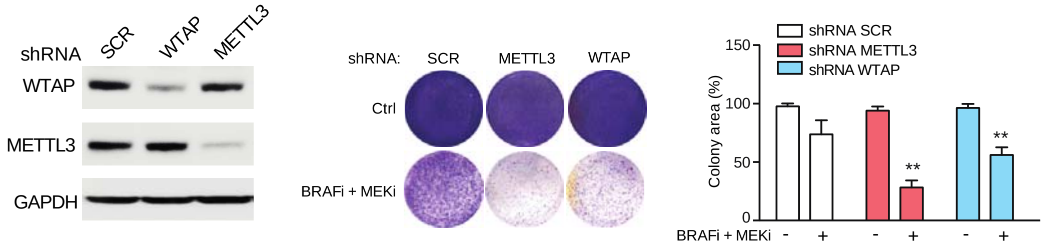

We then performed a polysome profiling of METTL3 shRNA-transduced persister cells . We extracted RNAs from each fraction and performed RT-qPCR to evaluate the effect of METTL3 knockdown on the translation of the candidate transcripts that are upregulated at the

translational level in persister cells. Knockdown of METTL3 inhibited the translation of several candidate transcripts upregulated at the translational level in persister cells, whereas minimal effects were observed on the translation of the HPRT housekeeping transcripts. These data further support the implication of m^{6}A modification in the translational reprogramming of melanoma persister cells. These results (shown below) have been added in Fig. 5d and have been discussed in the revised version of the manuscript.

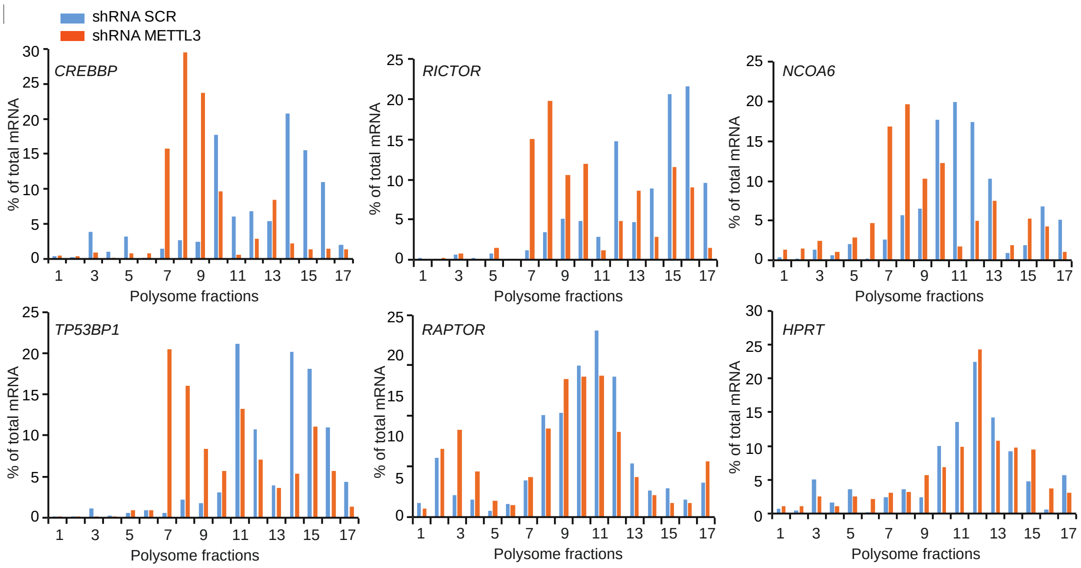

In addition, the authors should perform a simple m6A-seq experiment to prove that m6A is indeed enriched in the 5’ UTR of translationally-upregulated transcripts. Other than that, I fully support publication of this fine study.

Response: Following the reviewer’s suggestion, we performed m^{6}A-seq experiment on parental and persister cells to further explore the levels of m^{6}A in the 5’UTR of translationally up-regulated transcripts. Total and polysome-bound mRNAs from persister cells showed slightly higher m^{6}A peak frequency in 5’UTR or in CDS close to the 5’ end than those from parental cells as shown below. We have added this result (shown below) in Supplementary Fig 9c.

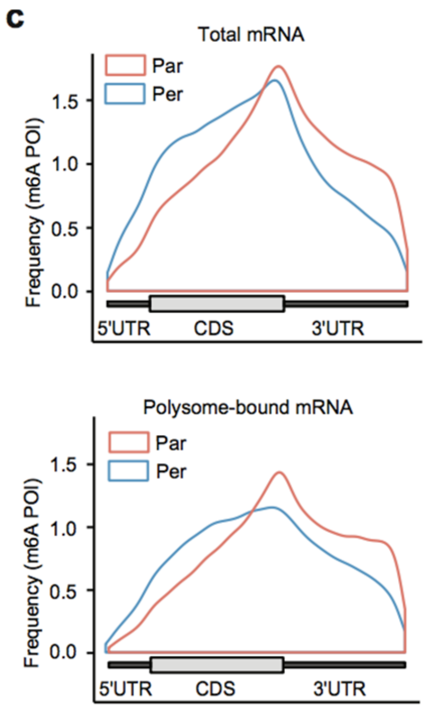

We found a relative enrichment of m^6A modification on the 5' end of the 178 translationally up-regulated transcripts as compared to the 180 most translationally down-regulated transcripts in total RNAs of both parental and persister cells (figure below). However, in mRNAs purified from polysomes, we observed higher 5'UTR m^6A modification of the 178 translationally up-regulated transcripts compared to the 180 most translationally down-regulated transcripts in persister cells, but not in parental cells. These results suggest that m^6A modification at 5'UTR of the 178 translationally up-regulated transcripts found in persister cells actually pre-existed in parental cells. It is possible that in proliferative parental cells, mRNA translation machinery is not selective whereas in quiescent persister cells, a decreased mRNA translation machinery may preferentially translate mRNAs harbouring m^6A-modified-enriched 5'UTR. This is consistent with our bioinformatics analysis of published datasets, showing higher 5'UTR m6A modification of the translationally up-regulated transcripts. In addition, decreased expression of ribosomal proteins and eIF4A1 were observed in persister cells (Supplementary Fig. 8e). Therefore, different subsets of mRNAs harbouring various m^6A distribution may send a signal to be translated when ribosomal resources are limited.

We have added theses new data (shown below) in the manuscript (Fig. 4f). We have also discussed these points in the results and discussion sections.

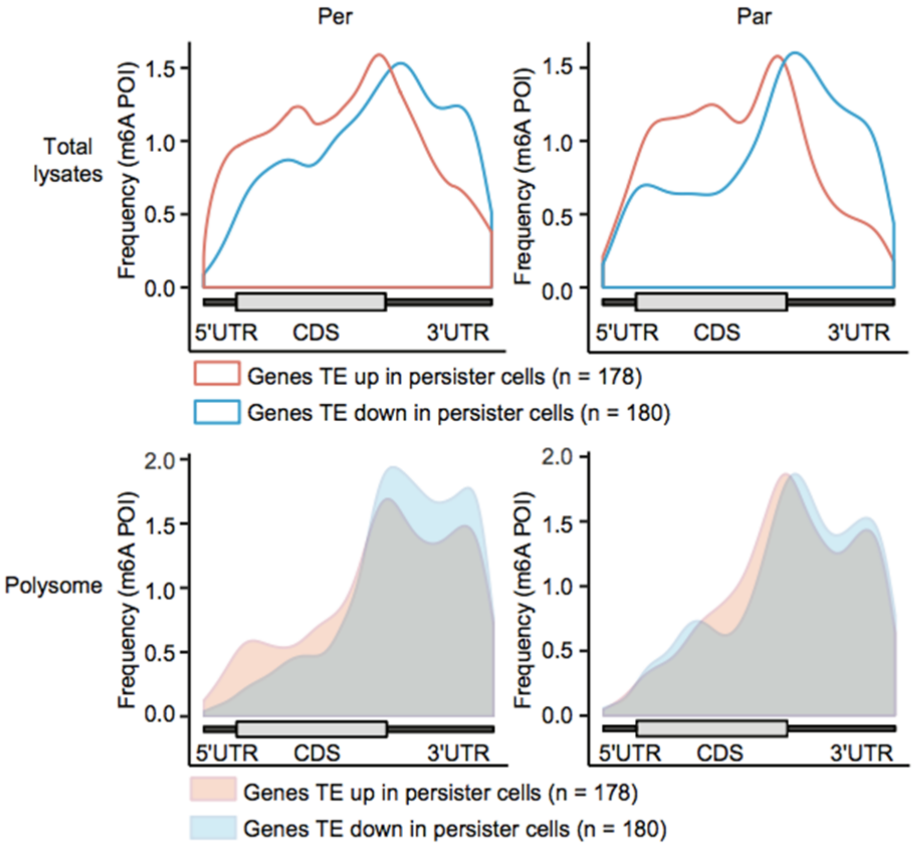

Per
Par
Total lysates
Polysome
Frequency (m6A POI)
0.0
0.5
1.0
1.5
2.0
5'UTR
CDS
3'UTR
Genes TE up in persister cells (n = 178)
Genes TE down in persister cells (n = 180)

Reviewer #3 (Remarks to the Author):

This manuscript ‘An epitranscriptomic mechanism underlies selective mRNA translation remodelling in melanoma persister cells’ by Shen et al., describes the changes in mRNA translation that accompany the short-term and reversible resistance of (BRAFV600 mutant) human cancer cells to pharmacological inhibition of the MAPK pathway (BRAFi/MEKi) and explores the underlying mechanism. The authors begin by describing and characterizing a subpopulation of ‘persister’ (Per) cells that can withstand MEKi. Next they find a global reduction in nascent polypeptide synthesis in Per cells that corresponds with a dramatic decrease in global translation as measured by polysome profiling using sucrose gradients. Next, they used microarrays to globally analyze changes in mRNA translation efficiency (TE) and identify that while many genes have lower TE in the Per cells compared to the parental (Par) control cells, there is a small subset of mRNAs that have increased TE in Per cells. This group of mRNAs is enriched for certain chromatin modifiers and stress-responsive kinases. shRNA-mediated depletion of 30 of these genes revealed that each of these individual genes has a modest impact on the frequency of PER cells. Testing a small panel of small-molecule inhibitors they found that Silvestrol, a known eIF4A inhibitor, selectively suppressed the growth of Per cells compared to Par cells. Moreover, expression of the mRNAs with TE upregulated in Per cells was found to be sensitive to Silvestrol. Interestingly the authors showed that eIF4Ai in combination with BRAFi/MEKi more effectively inhibited cancer cell viability (colony formation) that either inhibitor alone. Finally, the authors interrogate the features of the mRNAs with increased TE in Per cells in an attempt to understand how translation of this subset of mRNAs might be distinguished and regulated differently from bulk mRNAs in Per cells. They find no difference in 5’ UTR length, or minimum free energy between TE up- or down- regulated genes, and a very small difference in GC-content. However their re-analysis of published m6A meRIP-Seq data suggests that the mRNAs with increased TE are enriched for m6A marks in their 5’UTRs and the 5’-end of the CDS and have less m6A in their 3’ UTRs compared to either TE-down or total mRNAs suggesting that m6A might somehow be involved in the regulation of the TE-up mRNAs in Per cells. Overall, this is a very interesting study. While much of the work is performed to an excellent standard and the results convincing, there remain some important questions that need to be addressed prior to publication in Nature Communications. In particular the possible connection between m6a, eIF4A1, and increased TE is quite weak and needs further

verification.

Response: We are happy that the reviewer found our work very interesting and convincing and are thankful for his/her appreciation for our efforts to address the potential function of m6A in melanoma persister cells. We have addressed the remarks raised by the reviewer as follows:

Major concerns:

1) Figure 4C (and S9a-b). Are the apparent differences in the m6A distribution between the sets of mRNAs related to differences in the relative mRNA abundance? In other words is the mRNA abundance of the TE-up genes more or less than the rest of the mRNAs (or the TE downregulated mRNAs) and could this represent a technical artifact that might account for these differences in the distribution of m6A across the mRNA? Also, it is unclear why the authors picked only the top 10% downregulated mRNAs and not the entire group of TE-down for this analysis. This could be addressed with more bioinformatics analysis showing the read number (mean and distribution) for each of the gene sets.

Response: We thank the reviewer for his/her comments and suggestion. From the three published m^6A-seq datasets, exomePeak was used to calculate the overall methylation degree by dividing the m^6A IP reads by the sum of IP and input reads. This analysis was based on the hypothesis that all the m^6A IP reads at a particular location follow the similar Binomial distribution (Methods. 2014, 69(3):274-81; Bioinformatics. 2016, 32(12): i378–i385). ExomePeak computes the C test by parameterizing the overall methylation sites. Therefore, by taking into account the total number of reads in each sample, the differential distribution between the TE up and down regulated transcripts is not likely due to the read count variation. We then plotted the read count distributions of the TE-up gene set and TE-down gene set from the three published datasets and did not observe significant differences between the two gene sets as shown below.

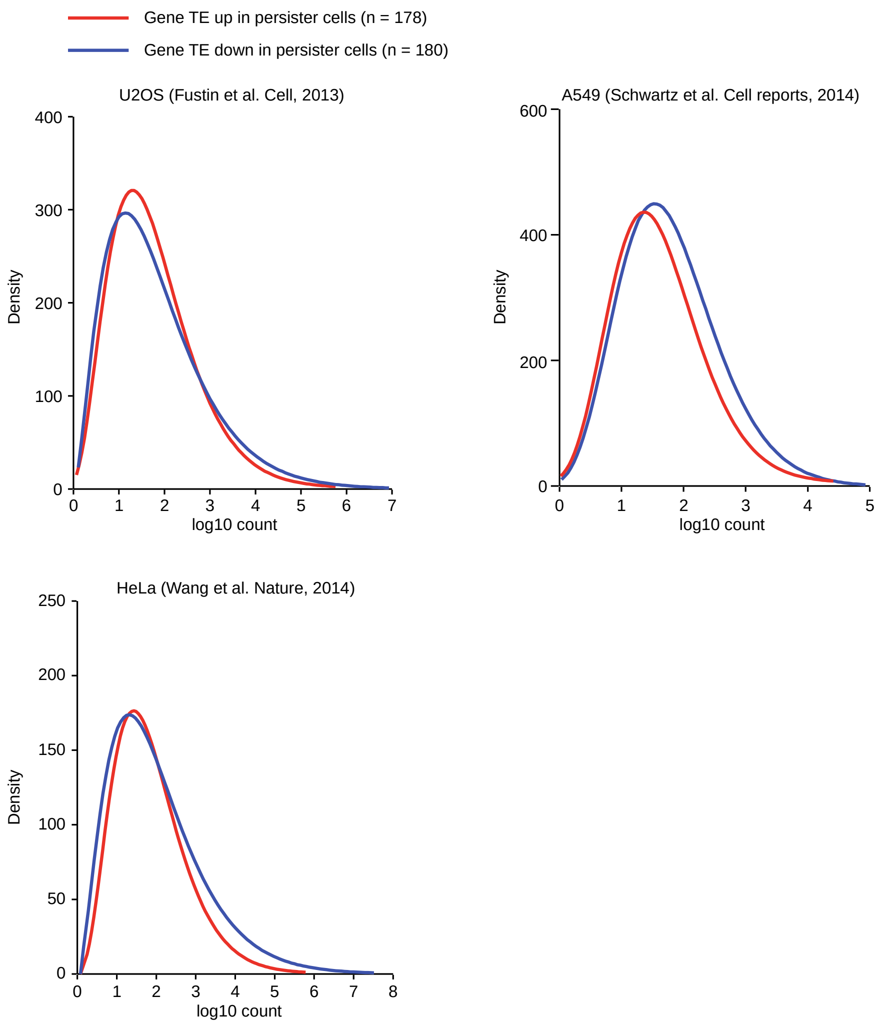

There are indeed many more TE-down regulated mRNAs than TE-up regulated mRNAs in persister cells. Among them, we chose to compare two gene sets that have the same relative proportion of transcripts with the translational change (73 %) versus homodirectional changes (27%). We have now clarified this in the revised manuscript.

2) Figure 4d and S9b. Considering the very dramatic decrease in the polysome peak it is surprising that only 1,287 genes were translationally downregulated more than 2-fold. I would have expected many more mRNAs with decreased TE in Per cells. Also, with this large decrease in polysome peak it is expected that a corresponding increase in 80S peak or at least in subpolysome fraction should be observed. Related to this, in Figure 4i there is big increase in the 80s peak in the Silvestrol-treated cells for both Par and Per cells. However the Polysome peak in Per (- Silv) is similar to Par (+ Silv), but the 80S peak in Per (- Silv) did not increase as much as Par (+ Silv). The most likely explanation for this result is that fewer Per

cells (or a lower concentration of cell lysate) was used for the polysome profiling of Per cells compared to the Par cells.

Response: Melanoma persister cells proliferate much slower than parental cells, explaining that ribosomal proteins and translation initiation factors are less abundant in persister cells compared to parental cells (Supplementary Fig 8e). Although the pool of available ribosomes and related translation factors is limited in persister cells, we show here, to address the reviewer's concern, that the ribosome composition in each fraction seems to remain stable between parental and persister cells.

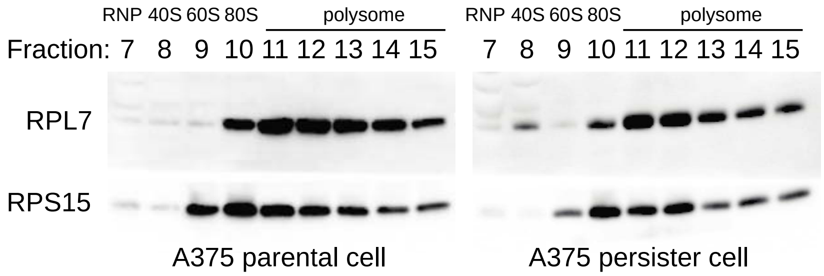

It was shown that a global reduction in ribosome/translation machinery levels altered more profoundly the translation of a selected subset of transcripts (Cell, 2018, 173: 90-103). This reduction in translation reflected by the polysome profile did not significantly increase the 80S peak (Cell, 2018, 173:90-103). In fact, inhibition of translation could be due to at least 4 main reasons: 1) global reduction in the levels of the translation machinery (*e.g.* the case for slow cycling persister cells, leading to the decreased levels of polysome and monosome); 2) inhibition in translation initiation (*e.g.* the case for silvestrol, leading to the accumulation of 80S); 3) inhibition of ribosome assembly (*e.g.* phosphorylation of eIF2a, leading to decreased levels of polysome and 80S); 4) inhibition of ribosome elongation, leading to the accumulation of 80S. In melanoma persister cells, our observations suggest that limited levels of translation machinery may be re-allocated to selectively translate transcripts harbouring 5'UTR m^6A modifications. In addition, the exon array analysis showed that a large number of transcripts (over 2865 genes) were less represented in the polysome fractions. In our analysis, we excluded the transcripts that decreased similarly in the input lysate and in the polysome fractions, in order to focus on the transcripts that were only altered at the translation level. We

have clarified the methods related to the critical points raised by the reviewer in the revised version of the manuscript.

3) In general dot blots are considered of limited use for relative m6A quantification (Figures 4e-j). Ideally, this would be done by HPLC/Mass Spec which is a more quantitative and reliable way of measuring m6A levels. Moreover, in these examples the MB-staining is barely visible and so it not possible to know whether there is equal loading of RNA on these blots. More importantly, the authors incorrectly conclude that the increased level of m6A in the polysome bound mRNAs in Per cells compared to the Par cells (Figure 4e). The statement in the results section that “A strong enrichment of m6A modification in mRNAs from heavy polysome fractions was found in persister cells compared to parental cells, with only a marginal increase observed in total mRNAs (Fig 4e)” does not appear to be the case since from the figure it seems that the relative ratio of m6A between Par and Per in the total RNA samples is quite similar to the m6A ratio in Polysome bound mRNAs from Par compared to Per cells. Therefore, the dot plot appears to show that the m6A level is increased in Per cells and that actively translating mRNAs harbor m6A marks but that the apparent increased m6A in polysomes simply reflects the increased m6A in the input RNAs from Per cells. It even seems that the increased m6A levels (in Per compared to Par) is very variable between different experiments (i.e. shown in figures 4e, figure 4i, and S9b). This further underscores my concerns about the dot blot assay for this type of analysis.

Response: Following the reviewer’s suggestion, we performed LC/MS-MS to quantify the m^6A level in parental and persister cells. This analysis allowed us to perform general analysis of different types of RNA methylation between parental and persister cells. Each level of RNA modification was normalized to the non-methylated form of adenosine or guanosine. Thereafter, RNA methylation from polysome-bound mRNAs was normalized to RNA methylation from total mRNAs. The data suggested that there is an enrichment of m^6A in polysome fractions from persister cells compared to parental cells at Day 1. On Day 9, this enrichment was reversed back to a level similar to that observed in parental cells. This phenomenon seems specific to m^6A modification since m^6Am, m^1A, Am and m^7G modifications did not show such enrichment in persister cells compared to parental cells at Day 1. These results (shown below) have been added in Fig. 4e.

- Persister cell Day 1 m^6A LC/MS-MS quantification plot:

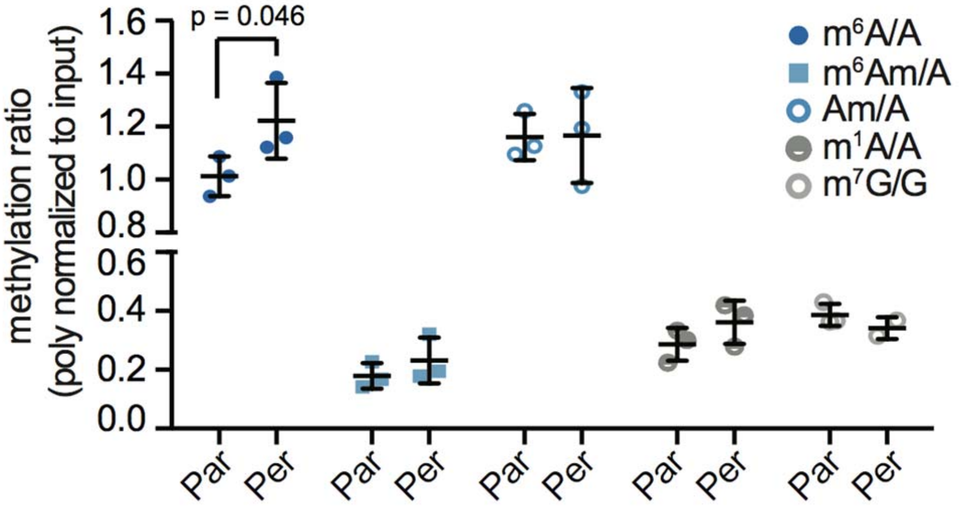

- Persister cell Day 9 (drug-free medium) m^{6}A LC/MS-MS quantification plot:

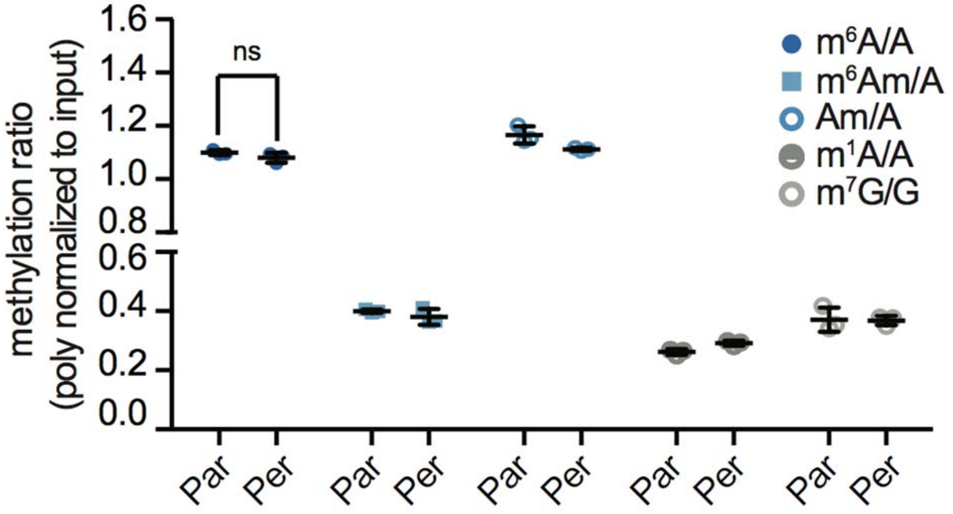

We also quantified the dot blot analysis and normalized the intensity of m^{6}A in mRNAs purified from polysome to the intensity of m^{6}A in total RNAs. Similar to the LC/MS-MS results, we observed an enrichment of m^{6}A level in persister cells compared to parental cells (shown below). Considering the concerns raised by the reviewer, we have moved the dot blot analysis in Supplementary Fig 9b. We also have discussed the results in the revised version of the manuscript.

- m^6A dot blot normalization plot:

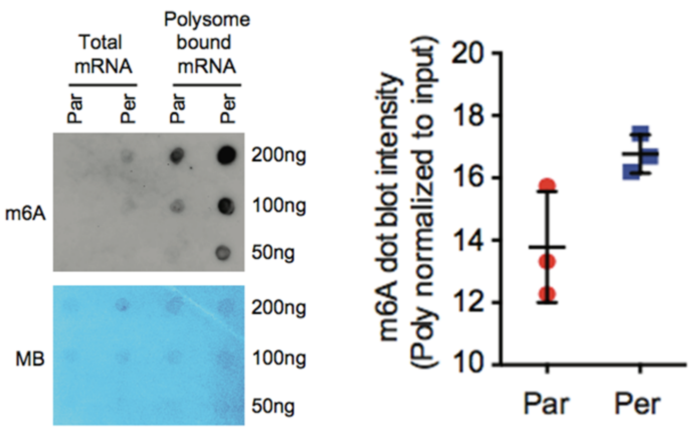

4) From the literature HPRT is reported to be a non-methylated mRNA and so presumably that is why the authors used this gene as a control (though this is not explained in the manuscript). If the authors picked HRPT as a non-methylated control mRNA, then why is the m6A enrichment shown for this mRNA in Fig 4f-j?

Response: We apologize that it was not clearly explained why we used this gene as control. We first chose HPRT as a control since it is a widely used house-keeping gene. Second, and most importantly, it was previously shown that its translation is independent of eIF4A regulation (*Nature. 2014, 513(7516):105-9*). Third, HPRT mRNA did not have m^6A peak from m^6A profiling data (*Nature, 2014,505:117–120*). Finally, its translation was shown not to be regulated by METTL3 (*Mol Cell, 2016, 62: 335-345*).

To clarify the calculation of m^6A enrichment, we have now detailed the normalization method in the figure legends and in the method section in the revised version of the manuscript.

The polysome-bound mRNAs were subjected to m^6A immunoprecipitation and RT-qPCR was performed with specific primers in m^6A-IP samples and input samples. Therefore, the m^6A enrichment of each mRNA candidate was calculated by normalizing m^6A-IP RT-qPCR to input RT-qPCR. Using HPRT as a non-m^6A modified transcript control, we did not observe any m^6A enrichment in m^6A-IP RT-qPCR quantification as shown now in Supplementary Fig 9d.

5) The m6A data are entirely correlative and no functional results are shown. Could the

authors try knocking down the m6A methyltransferase METTL3 and testing the affect on the frequency of Per cells and the expression of the TE-up (and control) genes?

Response: To add functional data, we knocked down METTL3 or WTAP by pLKO.1 shRNAs in A375 cells. We found that knockdown of METTL3 or WTAP strongly sensitizes melanoma cell exposure to BRAFi/MEKi. This result (shown below) has been added in Fig. 5a.

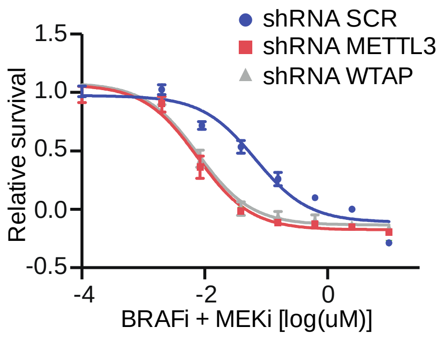

We subsequently showed that knockdown (shRNA) of either METTL3 or WTAP significantly reduced the persister cell-derived colony formation. These results (shown below) have been added in Fig. 5b, c and Supplementary Fig 10a.

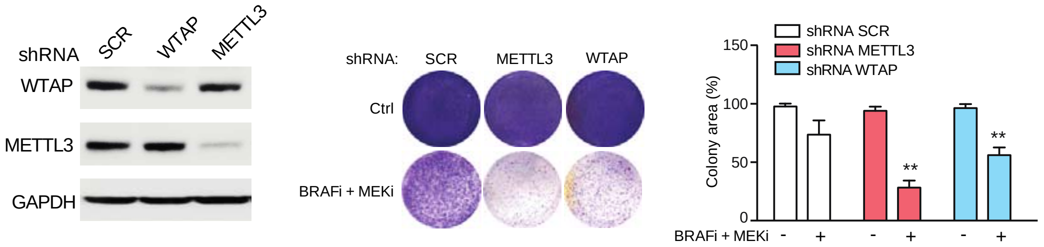

We then performed a polysome profiling of METTL3 shRNA-transduced persister cells. We extracted RNAs from each fraction and performed RT-qPCR to evaluate the effect of METTL3 knockdown on the translation of the candidate transcripts that are upregulated at the translational level in persister cells. Knockdown of METTL3 inhibited the translation of several candidate transcripts upregulated at the translational level in persister cells, whereas minimal effects were observed on the translation of the HPRT housekeeping transcripts. These data further support the implication of m^6A modification in the translational

reprogramming of melanoma persister cells. These results (shown below) have been added in Fig. 5d and have been discussed in the revised version of the manuscript.

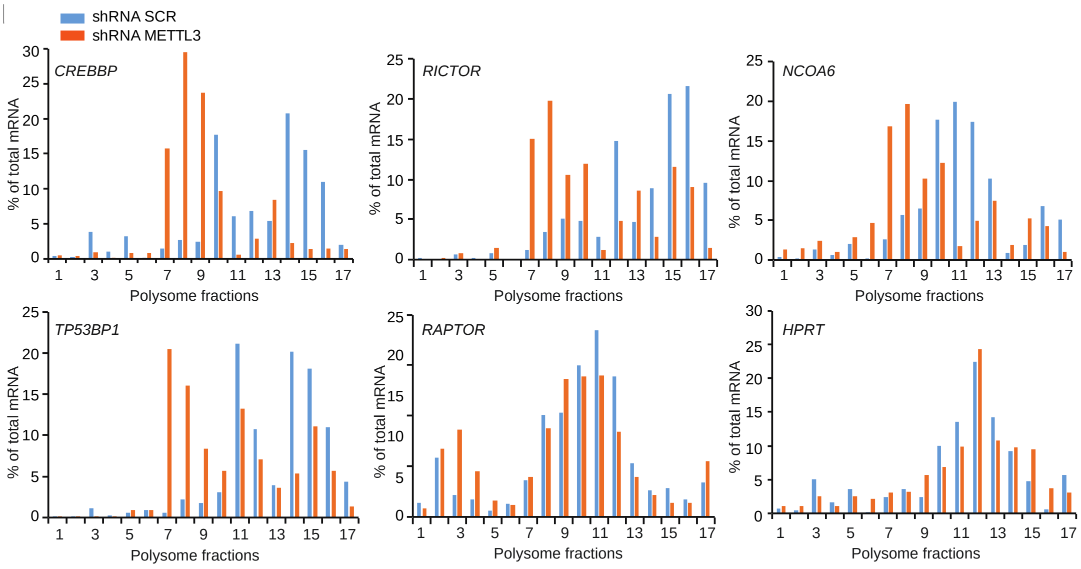

REVIEWERS' COMMENTS:

Reviewer #1 (Remarks to the Author):

The authors have thoroughly addressed all of my pressing comments.

Jeremy R Graff

Reviewer #3 (Remarks to the Author):

The authors haven done an admirable job in repsonspdng to the original comments. In particular the additional analysis of m6A in the parental and persister cells (Figure 4) and the new functional studies implicating METTL3 and WTAP in the resistance to MEKi (Figure 5) add substantial support to the authors' model and this exciting work is now suitable for publication in Nature Communications. It is likely to generate a lot of interest.
